# Low levels of ribosomal RNA partly account for the very high photosynthetic phosphorus-use efficiency of Proteaceae species

**DOI:** 10.1111/pce.12240

**Published:** 2013-12-23

**Authors:** Ronan Sulpice, Hirofumi Ishihara, Armin Schlereth, Gregory R Cawthray, Beatrice Encke, Patrick Giavalisco, Alexander Ivakov, StÉphanie Arrivault, Ricarda Jost, Nicole Krohn, John Kuo, Etienne Laliberté, Stuart J Pearse, John A Raven, Wolf-rüdiger Scheible, François Teste, Erik J Veneklaas, Mark Stitt, Hans Lambers

**Affiliations:** 1 Max Planck Institute of Molecular Plant PhysiologyAm Mühlenberg 1, Potsdam-Golm, D-14476, Germany; 2 School of Plant Biology, The University of Western Australia35 Stirling Highway, Crawley (Perth), Western Australia, 6009, Australia; 3 Centre for Microscopy and Microanalysis, The University of Western Australia35 Stirling Highway, Crawley, Western Australia, 6009, Australia; 4 Division of Plant Sciences, University of Dundee at JHI, James Hutton InstituteInvergowrie, Dundee, DD2 5DA, UK; 5 Plant Biology Division, The Samuel Roberts Noble Foundation2510 Sam Noble Parkway, Ardmore, OK, 73401, USA

**Keywords:** *Banksia*, carbon metabolism, delayed greening, glucose 6-phosphate, *Hakea*, PPUE, Rubisco, shikimate dehydrogenase

## Abstract

**Abstract:**

**Proteaceae species in south-western Australia occur on phosphorus- (P) impoverished soils. Their leaves contain very low P levels, but have relatively high rates of photosynthesis. We measured ribosomal RNA (rRNA) abundance, soluble protein, activities of several enzymes and glucose 6-phosphate (Glc6P) levels in expanding and mature leaves of six Proteaceae species in their natural habitat. The results were compared with those for *Arabidopsis thaliana*. Compared with *A*. *thaliana*, immature leaves of Proteaceae species contained very low levels of rRNA, especially plastidic rRNA. Proteaceae species showed slow development of the photosynthetic apparatus (‘delayed greening’), with young leaves having very low levels of chlorophyll and Calvin–Benson cycle enzymes. In mature leaves, soluble protein and Calvin–Benson cycle enzyme activities were low, but Glc6P levels were similar to those in *A*. *thaliana*. We propose that low ribosome abundance contributes to the high P efficiency of these Proteaceae species in three ways: (1) less P is invested in ribosomes; (2) the rate of growth and, hence, demand for P is low; and (3) the especially low plastidic ribosome abundance in young leaves delays formation of the photosynthetic machinery, spreading investment of P in rRNA. Although Calvin–Benson cycle enzyme activities are low, Glc6P levels are maintained, allowing their effective use.**

## Introduction

Australia in general (Beadle 1962), and south-western Australia in particular (McArthur 1991; Wyrwoll *et al.* 2014), is known for its severely nutrient-impoverished soils. Among the macronutrients, phosphorus (P) is the least available nutrient because of prolonged soil weathering and the low P content of the parent material (Lambers *et al.* 2010; Laliberté *et al*. 2012). Non-mycorrhizal Proteaceae are an important component of the vegetation on the most severely P-impoverished soils (Lambers *et al.* 2010, 2013; Hayes *et al.* 2014). Species in this family typically form cluster roots that effectively mine P by releasing large amounts of low-molecular weight carboxylates to release sorbed P from soil particles (Lambers *et al.* 2008b). It is also striking that mature leaves of Proteaceae species from south-western Australia exhibit relatively high rates of photosynthesis, despite their extremely low leaf P concentrations ([P]) (Wright *et al.* 2005; Denton *et al.* 2007; Lambers *et al.* 2012b). This contrasts with most other species, where P deficiency usually results in low rates of photosynthesis per unit leaf area (Brooks *et al.* 1988; Fredeen *et al.* 1990; Kirschbaum & Tompkins 1990). Because phosphate rock is a non-renewable resource (Gilbert 2009; Elser 2012), our aim was to enhance our understanding of the biochemical basis for the high photosynthetic P-use efficiency (PPUE) of Proteaceae species and explore if this trait is worth pursuing towards developing more P-efficient crops (Lambers *et al.* 2011; Veneklaas *et al.* 2012).

Studies on barley, *Arabidopsis thaliana*, spinach and other species that typically grow on relatively fertile soils have shown that the major organic P fractions in leaves are nucleic acids (40–60%), phospholipids (26–39%) and P-containing metabolites (20–30%) (Veneklaas *et al.* 2012). Ribosomal RNA (rRNA) comprises the major component of the nucleic acid fraction (Dyer *et al.* 1971; Raven 2012). The major P-containing metabolites include phosphorylated intermediates of glycolysis and the Calvin–Benson cycle, and free nucleotides and nucleotide sugars (Arrivault *et al.* 2009). Phospholipids are major membrane components (Jouhet *et al.* 2004; Andersson *et al.* 2005). In these species, a limiting P supply leads to a general decrease in the level of RNA (Hewitt *et al.* 2005), a decrease in the levels of sugar phosphates and nucleotides (Brooks *et al.* 1988; Hurry *et al.* 2000; Zrenner *et al.* 2006; Morcuende *et al.* 2007), and partial replacement of phospholipids by galactolipids (Dörmann & Benning 2002; Kelly *et al.* 2003). Phosphorus limitation also leads to the induction of specialised glycolytic enzymes that do not require nucleotides (Theodorou & Plaxton 1993; Plaxton & Tran 2011) and the induction of extracellular phosphatases (Del Pozo *et al.* 1999; Li *et al.* 2002; Plaxton & Tran 2011).

High rates of photosynthesis require a fine balance between the levels of free orthophosphate (Pi) and phosphorylated intermediates, with photosynthesis being inhibited when free Pi is depleted (Heldt *et al.* 1977, see Stitt *et al.* 2010 for a recent review, Stitt & Quick 1989). Orthophosphate is incorporated by the thylakoid ATPase into ATP, transferred to phosphorylated intermediates via the ATP-consuming reactions of the Calvin–Benson cycle, and released again during the synthesis of end-products like sucrose and starch (Heldt 2005; Stitt *et al.* 2010). In principle, a lower level of Pi, adenine nucleotides and phosphorylated intermediates might be compensated by having higher levels of enzymes. This would be analogous to the increase in enzyme activities that occurs at low temperature, and which partly compensates for the decrease in K_cat_ at low temperature (Stitt & Hurry 2002; Usadel *et al.* 2008). The levels of enzymes involved in sucrose synthesis increase markedly when *A. thaliana* is grown at a low P supply (Hurry *et al.* 2000; Ciereszko *et al.* 2001; Morcuende *et al.* 2007). Enzymes for sucrose synthesis also increase markedly, and the levels of Calvin–Benson cycle enzymes remain high or increase slightly in the *pho1* mutant, which is defective in transport of P to the shoot (Hurry *et al.* 2000). However, it should be noted that the synthesis and maintenance of high levels of enzymes will require investment of P in the protein synthesis machinery, which itself represents a major fraction of P in low-P leaves (Veneklaas *et al.* 2012).

None of the studies of acclimations to low P cited earlier addressed species that are adapted to extremely low P conditions. In a previous paper, we showed that following leaf expansion in six Proteaceae species growing in the field on severely P-impoverished soils in south-western Australia, phospholipids are much more extensively replaced by galactolipids and sulfolipids than in other species studied to date (Lambers *et al.* 2012b). For the same species, here, we present results on the abundance of rRNA, representative metabolites [starch, glucose 6-phosphate (Glc6P) ], and a set of enzymes involved in carbon fixation and central metabolism, which represent a major component of the protein pool in leaves. We compare the results for Proteaceae species growing in the field with those obtained on the model dicot *A. thaliana* grown at a range of controlled conditions in a growth room and in a greenhouse under P-sufficient or P-limited conditions. Previous research on *A. thaliana* prompted the hypothesis that P-efficient Proteaceae invest relatively little P in intermediates of carbon metabolism, and compensate by maintaining high levels of enzymes. However, we also consider the alternative possibility that the maintenance of high levels of enzymes may not be P-neutral, because this will require high rates of protein synthesis, and, hence, a larger investment of P in rRNA (Veneklaas *et al.* 2012).

## Materials and Methods

### Field site and Proteaceae species description

As before, we chose to test our hypotheses using plants growing at a location that is well-known for its high plant biodiversity (particularly of Proteaceae) and its ancient, nutrient-impoverished soils, Lesueur National Park in south-western Australia (Hopper & Gioia 2004; Mucina et al. 2014). The sites chosen were the same as those used for our recent study on replacement of phospholipids by other lipids during leaf development (Lambers et al. 2012b), with the exception of the site for *Hakea prostrata*, which had been burned since our previous measurements. For determination of leaf [N] in *H. prostrata*, we sampled both at the original site and at a nearby site that was not burned, finding no significant difference. Data on leaf morphological and chemical traits were collected for both young expanding and mature one-year-old leaves.

### Growth of *A. thaliana*

*Arabidopsis thaliana* plants (Col-0 accession) were grown under a range of controlled conditions at the Max Planck Institute of Molecular Plant Physiology (Potsdam, Germany), including conditions that decrease growth of P-replete plants and conditions that decrease growth because of low P supply. To decrease growth rates in P-replete plants, we used a short photoperiod. Seeds of the Col-0 accession were germinated and seedlings grown for one week in a 16 h light [150 *μ*mol photons m^−2^s^−1^, 20 °C, 75% relative humidity (RH) ] 8 h dark (6 °C, 75% RH) regime in a standard peat-vermiculite-sand (6:3:1) substrate (Stender AG, 15926 Luckau, Germany). After another week in an 8 h light (160 *μ*mol photons m^−2^s^−1^, 20 °C, 60% RH), 16 h dark (16 °C, 75% RH) regime, individual plants were transferred to pots (6 cm diameter) filled with either standard substrate, and placed for another nine days in a Percival AR-36 L2 growth chamber (Percival-Scientific, Perry, IA, USA) set to the different photoperiods tested. The pots were irrigated twice a week with deionized water. All analysed leaf samples were harvested on the same day and within one hour at the beginning and the end of the light period, by snap-freezing in liquid nitrogen (N). Plants were grown at a range of day lengths; the longer day lengths were used for comparison with Proteaceae species growing in their natural habitat, whereas the shorter ones were used to impose conditions where *A. thaliana* grows very slowly, which is typical for the investigated Proteaceae species. In one experiment, we also harvested leaves at four stages of leaf development from 33-day-old plants growing in an 8 h photoperiod.

To investigate the response of *A. thaliana* to a decrease in P supply, plants were grown under normal greenhouse conditions with average light intensity of 160 *μ*mol photons m^−2^s^−1^, 16 h light and 8 h dark regime (at the Max Planck Institute of Molecular Plant Physiology). Plants were divided into two sets: one set was supplied with a full-nutrient soil, using Osmocote® fertilizer, containing sufficient P (Pi = 15.5 mM), and the other one was supplied with less Osmocote® fertilizer (Pi = 1.1 mM). All analysed leaf samples were harvested between 6 and 8.5 h into the light period by snap-freezing in liquid N. Also, in the P-replete and P-deficient treatments, sets of leaves were harvested along the developmental gradient, in this case in three groups: young leaves (<1–9 mm length), mid-stage (9 mm to second largest), and mature leaves (largest to oldest leaves).

### Leaf N analyses

Mature leaves were harvested from plants of similar age and health condition as in Lambers *et al*. (2012b) from each of the six species (*Banksia attenuata* R.Br., *B. candolleana* Meisn., *B. menziesii* R.Br., *H. flabellifolia* Meisn., *H. neurophylla* Meisn., *H. prostrata* R.Br.). We collected 10 healthy mature leaves per plant from the top third of the crown from three replicate plants within a 100 m radius. Fresh weight (FW) was determined immediately after collecting, and then leaves were dried at 70 °C for 48 h, ground using a ball mill, and 100 mg analysed for total N following the Dumas method (Buckee 1994). N was quantified with an Elementar Vario Macro (Hanau, Germany) directly after combustion and analysis of the evolved gases.

### Enzyme and metabolite analyses

Leaves collected from the same plants as those previously used for gas-exchange measurements (Lambers *et al.* 2012b) were snap-frozen in liquid N immediately after field collection, with the exception of leaves used to assess variation in starch concentration from dawn till midday, which were collected in December 2012. Fresh weights were determined and the samples were transferred on dry ice to the Max Planck Institute of Molecular Plant Physiology. Approximately 200 mg of fresh material was ground to a fine powder and aliquoted (samples ca. 20 mg FW) with a cryorobot (Labman Automation, Stokeley, UK) for use in enzyme and metabolite analyses. The aliquots were stored at −80 °C until use. Enzyme extractions took around 1 min per sample, and enzyme activities were measured as in Gibon *et al*. (2004, 2009) and Sulpice *et al.* 2010, 2007) using the dilutions and incubation times indicated in the Supporting Information Table S1.

For measurements of metabolites and structural components, assays were performed following ethanol extraction at high tissue dilution, using 0.08, 0.2, 0.06 and 0.08 mg FW per assay for soluble proteins, starch, amino acids and the major P-containing small metabolite Glc6P, respectively (Gibon *et al.* 2009; Sulpice *et al.* 2009). Glc6P occupies a central position in metabolism, being involved in the pathways of sucrose and starch synthesis, sucrose and starch degradation, cell wall synthesis, and glycolysis. Recoveries were determined as described for enzyme activities (see Supporting Information Table S1) and for proteins, starch, amino acids and Glc6P were 86, 107, 88 and 81%, respectively. Measurements were only possible for abundant metabolites that could be detected with great sensitivity, because of interfering substances in the extracts. This problem could not be circumvented by preparation of extracts with trichloroacetic acid for measurement in dual wavelength photometers.

We measured a set of enzymes from the Calvin–Benson cycle, starch synthesis, sucrose metabolism, glycolysis, organic acid metabolism (fumarase) and amino acid biosynthesis in immature and mature leaf material from the six Proteaceae species (see Supporting Information Table S1 for information on the enzymes and abbreviations). The reliability of the extraction and assay was checked by preparing a powder from six *Banksia* and *Hakea* species, and a standard powder prepared from greenhouse-grown *A.* *thaliana*, extracting the powders separately or after mixing them in a 1:1 ratio, and determining enzyme activities in each extract. Recoveries of enzyme activities were 78–130% (Supporting Information Table S1). This effective recovery of enzyme activity is remarkable, because the Proteaceae leaves contain large amounts of gums and other secondary metabolites that complicate many biochemical and molecular analyses. The good recovery of enzyme activities is probably due to the high sensitivity of the enzyme assays used in this study (Gibon *et al.* 2004). This allows extracts to be prepared at high dilution and used at even higher dilution in the enzyme assays. Rubisco was assayed immediately after extraction, and after incubation with a high CO_2_ concentration at pH 8 to fully carbamylate the lysine in the active site.

### Extraction of RNA, cDNA synthesis and quantification of rRNA

Ribosomal RNA represents a major pool of P in organic compounds (see Introduction). Therefore, we used methods that were previously developed to quantify ribosome abundance in *A. thaliana* (Piques *et al.* 2009; Pal *et al.* 2013) to determine ribosome abundance in immature and mature leaves of the Proteaceae species. Briefly, RNA is extracted, a mixture of oligo (dT)_20_ primers and random hexamers are used to generate cDNA, and qRT-PCR is then used to measure the levels of cytosolic 18SrRNA and plastidic 16SrRNA, using specific primer pairs for each RNA species. To allow reliable quantification, a set of six external RNA species were added at different concentrations to the powdered plant material before extraction. These external standards were used to control for differential RNA loss during extraction and differential efficacy of amplification of RNA to cDNA in different plant material. Ribosome number was calculated by determining the amounts of the small subunits of cytosolic and plastidic rRNAs by qRT-PCR, and we used these to calculate plastidic and cytosolic ribosome abundance, assuming that each ribosomal RNA corresponds to one ribosome (Piques *et al.* 2009; Pal *et al.* 2013). Absolute quantification of total rRNA species was performed as in Piques *et al*. (2009), with some modifications.

One milligram FW aliquots of Proteaceae leaf material were homogenized with 800 *μ*L of RNA extraction buffer [1 M Tris/HCl, 1% (v/v) SDS, 10 mM ethylenediaminetetraacetic acid, 2% (w/v) PVP, 2% (w/v) PVPP, and 0.5% (v/v) *β*-mercaptoethanol] and spiked with a mix of the six artificial Poly(A) + RNAs (Ambion/Life Technologies GmbH, Darmstadt, Germany) in the dynamic range 9.6 × 10^12^ to 3.75 × 10^10^ copy number per gram FW. After centrifugation at 12 000 *g* for 2 min, the supernatant was transferred, mixed with an equal volume of phenol/chloroform/isoamylalcohol (25/24/1) and then centrifuged at 12 000 *g* for 15 min. The aqueous phase was transferred to a new tube and was again mixed with phenol/chloroform/isoamylalcohol (25/24/1). This step was repeated until the interface appeared clear. The aqueous phase was then transferred to a new tube and mixed with chloroform/isoamylalcohol (24:1, v/v) and centrifuged at 12 000 *g* for 15 min. The aqueous phase was transferred and mixed with 3 M Na acetate (10/1, v/v) and 100% ethanol (1/2.5, v/v), and then incubated at −80 °C for 30 min. The RNA was then pelleted by centrifugation at 12 000 *g* for 30 min and the pellet dissolved in 200–400 *μ*L water. A solution of LiCl (4 M) was added (v/v) and the mix stored at −4 °C overnight. The mix was then centrifuged at 4 °C for 10 min at 15 682 *g*, the supernatant discarded and the pellet washed with 500 *μ*L of 2 M LiCl. Finally, the pellet was rinsed with 500 *μ*L of 70% (v/v) ethanol, air dried and dissolved in 30 *μ*L of RNAse-free water. The cDNA was synthesized with 5–50 ng of total DNase I-treated RNAs, a mixture of oligo-d(T)_20_ primers (100 ng) and random hexamers (0.1 nmol) using the SuperScript III First-Strand Synthesis System (Invitrogen/Life Technologies GmbH, Darmstadt, Germany), according to the manufacturer's instructions. qRT-PCR reactions were performed in a volume of 10 *μ*L with 1/500, 1/50 and 1/5 dilutions of the cDNAs and 200 nM of each gene-specific primer. Power SYBR Green PCR Master Mix (Applied Biosystems/Life Technologies GmbH, Darmstadt, Germany) was used to monitor double-strand DNA synthesis. Standard curves for the six spike-in controls always had *R*^2^ values > 0.98. The threshold cycle (CT) values and slopes were similar in the various Proteaceae species to those obtained with *A. thaliana* (Supporting Information Table S2), confirming that the RNA extraction, cDNA synthesis and qRT-PCR reactions were not inhibited in extracts from the Proteaceae species, and validating the quantification of rRNA copy number. The external standards also provided an internal calibration, which was used to convert CT units into rRNA copy number. Standard curves were used to calculate the abundance (copy per gram FW) of the cytosolic and plastidic ribosomes. The primers used to amplify the genes for the cytosolic, and plastidic small subunit rRNAs were designed using *Hakea* rRNA sequence, as in Piques *et al*. (2009) and Pyl *et al*. (2012). All primers are listed in Supporting Information Table S3.

For comparison, we analysed cytosolic and plastidic rRNA at four stages of leaf development in 33-day-old P-replete *A. thaliana* growing in an 8 h light/16 h dark cycle (irradiance 150 *μ*mol m^−2^s^−1^). Four groups of leaves were harvested corresponding to the oldest four leaves (group a), all other leaves with a length >9 mm (group b), all leaves between 4 and 9 mm (group c) and all leaves with a length <4 mm (group d). It is difficult to specify a maximum size for *A. thaliana* leaves, as there is a strong tendency for successive leaves to attain a larger final size. The largest leaves in these rosettes at the time of harvest were about 17 mm long, indicating that leaves <9 and <4 mm have attained less than 53 and 23% of their final length, respectively. Physiologically, these sets of leaves approximately correspond to (1) mature leaves; (2) leaves that are still slowly expanding; (3) rapidly expanding leaves; and (4) leaves in the early stage of expansion growth or the cell division stage (Baerenfaller *et al.* 2012). The average size of leaves in groups c and d is about 20 and 4% of the final leaf size, respectively. Estimates of leaf expansion rates in comparable plants were 0.012, 0.114, 0.362 and 0.528 mm^2^ mm^−2^ day^−1^ (Supporting Information Table S4).

### Statistical analysis

Differences between young and mature leaves of Proteaceae for different metabolites and enzyme activities were tested using linear mixed-effect models (Pinheiro & Bates 2000), with random intercepts per species. To test for differences between mature leaves of Proteaceae and *A. thaliana* leaves, we firstly averaged the data for each Proteaceae species as well as for each photoperiod level (for *A. thaliana*), and then used linear models (Pinheiro & Bates 2000) on these averaged data. Residuals were visually inspected for heteroscedasticity and particular error structures were specified if they significantly improved the models, as evaluated via likelihood ratio tests (Pinheiro & Bates 2000). Analyses were conducted in the R Environment (R Development Core Team 2012), using the ‘nlme’ package (Pinheiro & Bates 2000).

## Results

### Leaf structure

**Figure 1:**
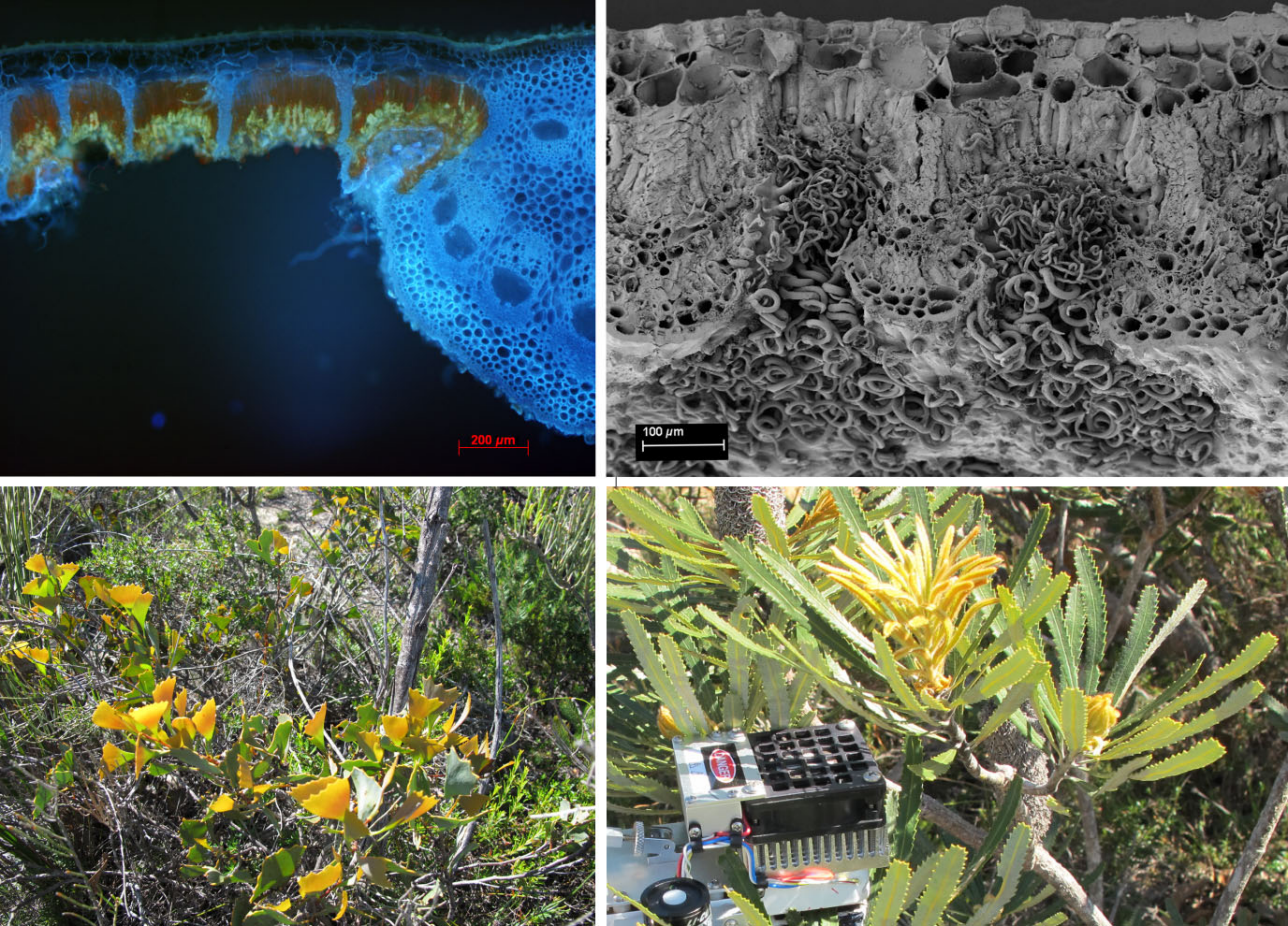
Anatomy and morphology of selected Proteaceae species used in this study. Top left: Cross section of a mature leaf of *Banksia menziesii*, showing a double epidermis, stomatal crypts, mesophyll (fluorescing red), sclerenchyma and a thick midrib. Top right: Cross section of an old leaf of *B. menziesii*, showing a double epidermis, stomatal crypts that are covered with trichomes, mesophyll, sclerenchymatic fibres and vascular tissue. Bottom left: Habitat photo of *Hakea flabellifolia*, showing dark green previous years’ foliage and yellow young expanding leaves (photo: Marion L. Cambridge). Bottom right: Habitat photo of *Banksia attenuata*, showing dark green previous years’ foliage and yellow young expanding leaves; both were used for gas-exchange measurements presented in Lambers *et al*. (2012b) (photo: Marion L. Cambridge).

Leaves of all six Proteaceae species showed very high values for leaf mass per unit area (LMA) and leaf dry matter content (LDMC) compared with those of *A. thaliana* (Table 1). This is accounted for by the large proportion of sclerenchymatic tissue in the leaf blade as well as a very thick midrib (Fig. 1). The leaf of *B. menziesii* had a double epidermis (Fig. 1), which is common for this genus (Swart 1988; Edwards *et al.* 2000; Mast & Givnish 2002; Jordan *et al.* 2005). Leaf thickness was calculated from these primary data, showing values for young leaves of 222–600 *μ*m and for mature leaves of 504–780 *μ*m, in the same range as described based on microscopic observations (Hassiotou *et al.* 2009; Lambers *et al.* 2012b) or using digital callipers (Hassiotou *et al.* 2010).

**Table 1:** LMA, LDMC and information on chemical composition of young, expanding leaves and fully expanded, mature leaves of *Banksia* and *Hakea* species growing in their natural habitat

Species	Leaf developmental stage	LMA (g DW m^−2^)	LDMC (g DW g^−1^ FW)	Leaf thickness (*μ*m)	Leaf [N] (mg g^−1^ DW)	Leaf [P] (mg g^−1^ DW)	Leaf N:P	Chlorophyll a/b ratio
*B. attenuata*	Young	195 ± 3	0.40 ± 0.01	488	ND	0.43 ± 0.013	ND	0.9
*B. attenuata*	Mature	310 ± 3	0.60 ± 0.01	517	9.2 ± 0.08	0.15 ± 0.020	60.5	1.8
*B. candolleana*	Young	240 ± 8	0.40 ± 0.01	600	ND	0.48 ± 0.032	ND	1.1
*B. candolleana*	Mature	468 ± 15	0.60 ± 0.02	780	7.5 ± 0.04	0.20 ± 0.013	38.2	2.0
*B. menziesii*	Young	224 ± 4	0.38 ± 0.01	589	ND	0.37 ± 0.039	ND	1.1
*B. menziesii*	Mature	345 ± 4	0.55 ± 0.02	627	8.3 ± 0.06	0.20 ± 0.008	40.6	2.3
*H. flabellifolia*	Young	181 ± 12	0.41 ± 0.06	441	ND	0.48 ± 0.101	ND	1.3
*H. flabellifolia*	Mature	315 ± 152	0.45 ± 0.04	700	11.8 ± 0.05	0.12 ± 0.018	32.3	2.3
*H. neurophylla*	Young	168 ± 12	0.38 ± 0.01	442	ND	0.38 ± 0.052	ND	1.2
*H. neurophylla*	Mature	371 ± 10	0.59 ± 0.00	629	8.0 ± 0.11	0.15 ± 0.014	53.3	2.7
*H. prostrata*	Young	100 ± 2	0.45 ± 0.04	222	ND	0.86 ± 0.046	ND	1.5
*H. prostrata*	Mature	272 ± 10	0.54 ± 0.01	504	12.7 ± 0.12	0.29 ± 0.005	44.4	2.7
*Arabidopsis thaliana*	Mature leaves	17.4 ± 0.4	0.08 ± 0.00	212	75 ± 0.26	4.7 ± 0.3	16	3.9

Mature leaves were produced in the preceding year, but were not senescent, because leaves of the investigated species continue to function for two years or more. Values are means ± SE (*n* = 6; or *n* = 3 for leaf [N] ). Leaf thickness was calculated from the values of LMA and LDMC (Lambers *et al.* 2008a). Data on leaf [P] in Proteaceae species are from Lambers *et al*. (2012b). Data for *A. thaliana* on LMA, LMA and leaf thickness are for plants grown at a 12 h day length under conditions identical to those used for the present plants; data on leaf [N] are from the literature (Tschoep *et al.* 2009). In a separate study where *Arabidopsis* plants were grown in long days (14 h/10 h) in a glasshouse, leaf protein was measured for both young (19.2 ± 0.4 mg g^−1^ FW) and old (14.9 ± 0.9 mg g^−1^ FW) leaves; for further data on leaf developmental stage-dependent changes in protein concentration in *Arabidopsis*, see Supporting Information Table S4. DW, dry weight; FW, fresh weight; LMA, leaf mass per unit area; LDMC, leaf dry matter content; N, nitrogen; ND, not determined; P, phosphorus.

Morphological features like metabolically inactive sclerenchymatic tissues and large epidermal cells (Fig. 1) areimportant to take into account when expressing metabolic parameters on a dry or FW basis. We therefore expressed many of the measured metabolites, processes or activities on an area or protein basis. Information about the rates of photosynthesis in this material was published in Lambers *et al*. (2012b). Briefly, the average rates of photosynthesis of Proteaceae species in ambient conditions in the field are very low in young expanding leaves (average 3.3 and range −4.1 to +12.8 *μ*mol CO_2_ m^−2^s^−1^), and high in mature leaves (10–23 *μ*mol CO_2_ m^−2^s^−1^). The rates of mature leaves are approximately 7 *μ*mol CO_2_ m^−2^s^−1^ in *A. thaliana* (Eckardt *et al.* 1997). Thus, all Proteaceae species photosynthesize more rapidly than *A. thaliana* on an area basis. Comparison with the leaf structural information (Table 1) indicates that the mature leaves of the *Banksia* and *Hakea* species have, on average, similar rates of photosynthesis on a FW basis (29 nmol CO_2_ g^−1^s^−1^ compared with 33 nmol CO_2_ g^−1^s^−1^ in *A. thaliana*).

### Ribosome abundance

Ribosomal RNA represents a major pool of P in organic compounds in plant leaves (see Introduction), with about half being in cytosolic and half in plastidic ribosomes (Piques *et al.* 2009; Pal *et al.* 2013). We investigated the levels of ribosomes, using methods that allowed us to distinguish between cytosolic and plastidic ribosomes. rRNA abundance was analysed in the same immature and mature leaves of the Proteaceae species that were used to measure metabolites, protein and enzyme activities (see later). For comparison, we analysed cytosolic and plastidic rRNA at four stages of leaf development in 33-day-old P-replete *A. thaliana* growing in an 8 h photoperiod. The sampled immature leaves of the Proteaceae species were approximately 50% of the final size. The groups a, b, c and d leaves of *A. thaliana* were 60% (fully expanded leaves, note that the size of the first fully expanded leaves is smaller than that of subsequent leaves; data not shown) 78, 20 and 4% of the area of the largest leaf in the rosette; this leaf being among group b leaves (see below for further discussion).

**Figure 2:**
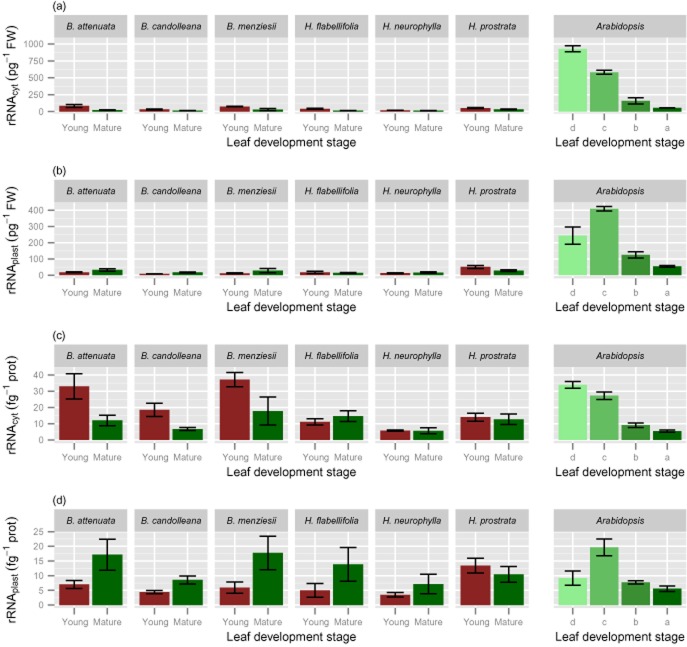
Ribosome copy number of young, expanding (red bars) and fully mature (>1 year old; green bars) leaves of six Proteaceae species growing in their natural habitat and of leaves at three stages of leaf development of *Arabidopsis thaliana* grown in a growth room at a day length of 8 h. Data for leaves of *A. thaliana* are arranged from youngest (palest green, d) to oldest (darkest green, a), representing: group d, leaves with a length < 4 mm; group c, leaves with a length between 4 and 9 mm; group b, leaves with a length > 9 mm, with the exception of the four oldest leaves; group a, oldest four leaves. Different leaf developmental stages are indicated along the *x*-axis. Values are means± SE (2 ≤ *n* ≤ 6). Leaves of the Proteaceae species were harvested during mid-morning; those of *A. thaliana* one hour before the end of the light period (a) cytosolic ribosome copy number per unit FW; (b) plastidic ribosome copy number per unit FW; (c) cytosolic ribosome copy number per unit soluble protein (d) plastidic ribosome copy number per unit soluble protein.

Ribosome abundance (on a FW basis) was much lower in the Proteaceae species than in *A. thaliana* (Fig. 2a, Supporting Information Table S5. The Proteaceae species contained two to four times less ribosomes than *A. thaliana* did in mature leaves: 2.96 × 10^13^ to 6.15 × 10^13^ ribosome g^−1^ FW (or 29.6–61.5 ribosomes pg^−1^ FW), compared with 11 × 10^13^ ribosomes g^−1^ FW (or 110 ribosomes pg^−1^ FW), respectively (Fig. 2a,b). The difference in ribosome abundance between the Proteaceae species and *A. thaliana* were strikingly larger in immature leaves (10–40 times).

Ribosome abundance showed a different developmental gradient in the Proteaceae species than in *A. thaliana*. Total ribosome abundance was the same (*H. prostrata*, *B. candolleana*) or only slightly higher (1.4- to 1.5-fold in *H. neurophylla*, *B. menziesii* and *H. flabellifolia*, and twofold in *B. attenuata*) in young expanding leaves than in mature leaves (Fig. 2a). This contrasts with *A. thaliana* leaves (Fig. 2a), which showed much higher ribosome numbers in younger leaves. When the oldest five leaves (stage a) are taken as the basis for comparison, ribosome abundance increased by 2.6-, 9.3- and 10.9-fold in leaf groups b, c and d, respectively (corresponding to leaves that are expanding slowly, expanding rapidly or are in the cell division or early expansion stages). The increase in ribosomes in leaf groups c and d is still more than three times and more than four times higher, when they are normalized on leaf group b.

There were also differences in the developmental gradients of cytosolic and plastidic ribosome abundance between *A. thaliana* and the Proteaceae species (Fig. 2a,b). In mature leaves, the ratio between plastidic and cytosolic ribosomes was close to unity in all of the Proteaceae species and in *A. thaliana* (see also Piques *et al.* 2009; Pal *et al.* 2013). In *A. thaliana*, a ratio close to unity was also found for group b and group c leaves, decreasing to about 0.3 in the youngest leaf stage (group a). The abundance of plastidic ribosomes per g FW was highest in the rapidly expanding leaves (group c), which contained eight times more plastidic ribosomes on a FW basis than mature leaves. Even the very young leaves of group d contained four times more plastidic ribosomes per unit FW than mature *A. thaliana* leaves. Based on their size relative to mature leaves, the immature Proteaceae leaves that we sampled in the field are best compared with the group b and c leaves of *A. thaliana*.

In most of the Proteaceae species the plastidic ribosome:cytosolic ribosome ratio decreased in immature leaves (to ∼0.5 in *H. flabellifolia* and *H. neurophylla*; ∼0.25 in *B. candolleana*, *B. menziesii* and *B. attenuata*), remaining close to unity only for *H. prostrata*). These data point to a very low investment in plastidic ribosome abundance in immature leaves in the Proteaceae species compared with that in immature *A. thaliana* leaves.

### Leaf protein and N concentrations

**Figure 3:**
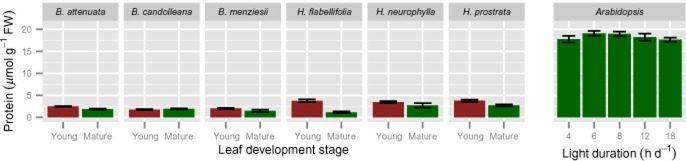
Leaf soluble protein concentrations of young, expanding (red bars) and fully mature (>1 year old in Proteaceae species; green bars) leaves of six Proteaceae species growing in their natural habitat and in *Arabidopsis thaliana* grown in a growth room at a range of day lengths, as indicated along the *x*-axis. Leaves of the Proteaceae species were harvested during mid-morning; those of *A. thaliana* at the end of the day.

Since rates of protein synthesis scale with rRNA content (Elser *et al.* 1996; Matzek & Vitousek 2009), we expected the low investment in rRNA in Proteaceae species to be associated with low levels of soluble protein and total N. Indeed, total soluble protein concentrations were five- to 10-fold lower in mature and immature leaves of the Proteaceae species than in *A. thaliana* leaves (Fig. 3). We suspect that there are also considerable amounts of protein in the cell wall of the Proteaceae species that would not be extracted by our methods (Albenne *et al.* 2013). As expected, mature leaf N concentrations of the plants analysed in the present study were also low: 7.5–11.8 mg g^−1^ dry weight (DW) (Table 1), but somewhat higher than values reported for leaves of *B. attenuata* and *B. menziesii*: 5.5 and 5.4 mg N g^−1^ DW, respectively (Tassone & Majer 1997). A low N concentration is typical for Proteaceae from south-western Australia (Lambers *et al.* 2012a), and compares with typical minimum and maximum vales of 8.7 ± 0.6 and 40.9 ± 5.7 mg N g^−1^ DW for 111 C_3_ species (Reich *et al.* 1997). The corresponding value in *A. thaliana* plants grown in the same conditions as used in this study is 75 mg N g^−1^ DW (Table 2; Tschoep *et al.* 2009).

**Figure 4:**
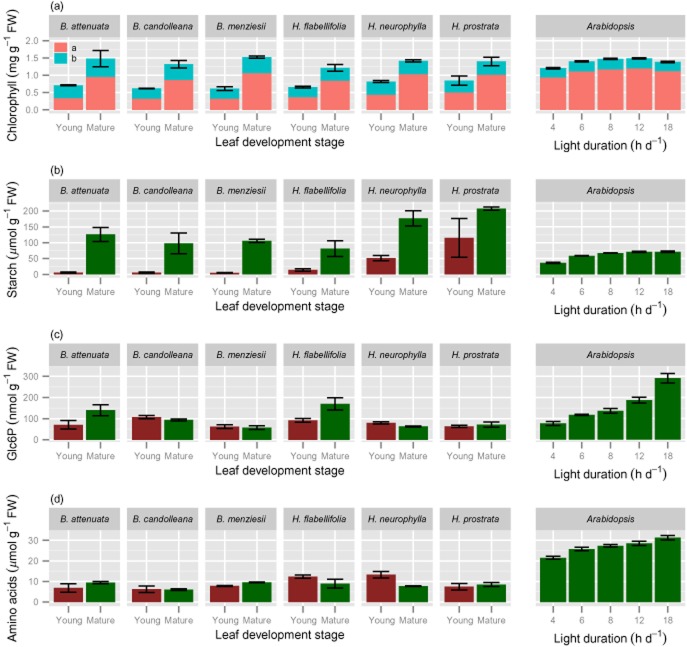
Leaf chemical composition of young, expanding (red bars, except for chlorophyll) and fully mature (>1 year old; green bars, except for chlorophyll) leaves of six Proteaceae species growing in their natural habitat and in *Arabidopsis thaliana* grown in a growth room at a range of day lengths, as indicated along the *x*-axis. Values are means ± SE (2 ≤ *n* ≤ 6). Leaves of the Proteaceae species were harvested during mid-morning; those of *A. thaliana* at the end of the day; those of *A. thaliana* at the end of the day. (a) Chlorophyll a and chlorophyll b concentrations; (b) starch concentrations (expressed as glucose equivalents); (c) Glc6P concentrations; (d) amino acid concentrations.

**Figure 5:**
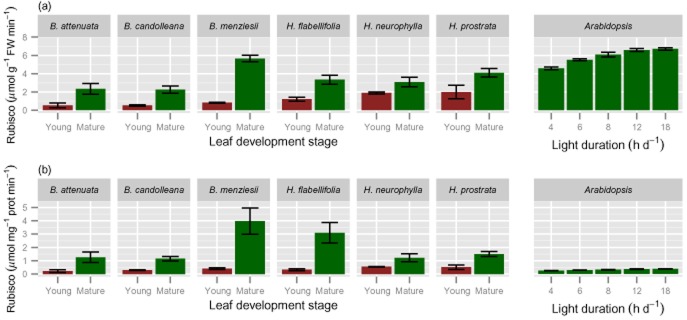
*In vitro* total Rubisco activities of young, expanding (red bars) and fully mature (>1 year old; green bars) leaves of six Proteaceae species growing in their natural habitat and in *Arabidopsis thaliana* grown in a growth room at a range of day lengths, as indicated along the *x*-axis. Values are means ± SE (2 ≤ *n* ≤ 6). Leaves of the Proteaceae species were harvested during mid-morning; those of *A. thaliana* at the end of the day. Rubisco was assayed after incubation with high CO_2_ concentration at pH 8 to fully carbamylate the lysine in the active site; (b) total Rubisco activity expressed per unit soluble leaf protein.

**Figure 6:**
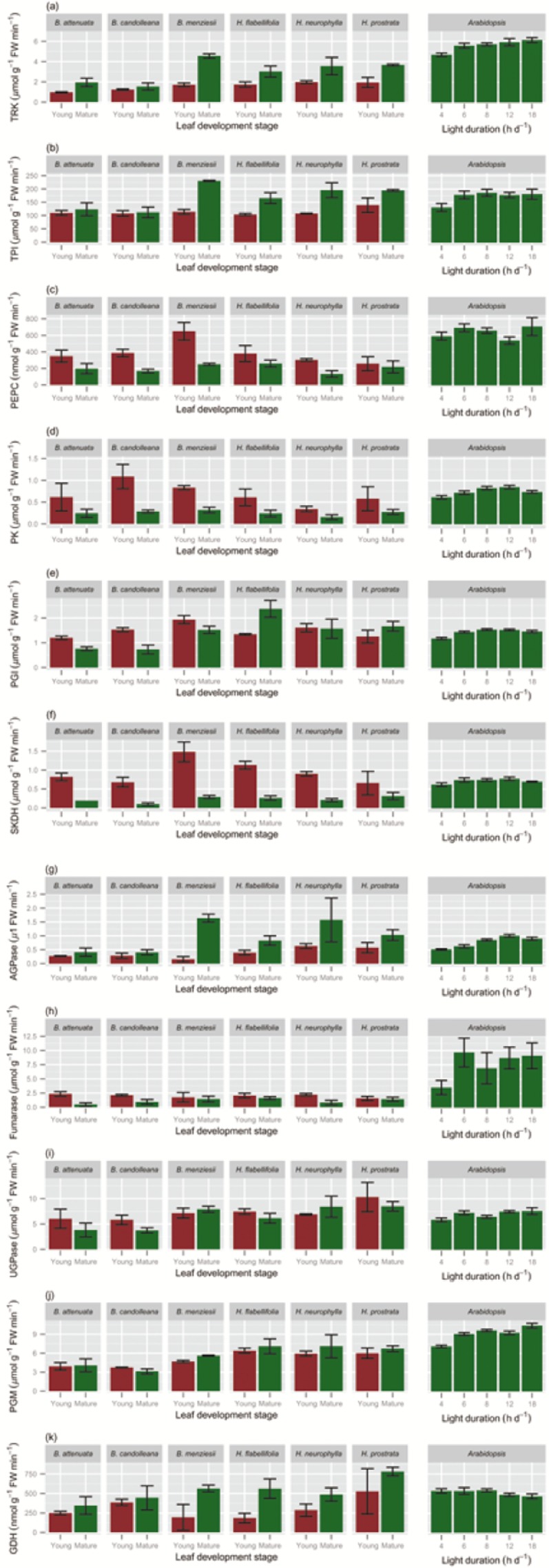
*In vitro* enzyme activities of young, expanding (red bars) and fully mature (>1 year old; green bars) leaves of six Proteaceae species growing in their natural habitat and in *Arabidopsis thaliana* grown in a growth room at a range of day lengths, as indicated along the *x*-axis. Values are means ± SE (2 ≤ *n* ≤ 6). Leaves of the Proteaceae species were harvested during mid-morning; those of *A. thaliana* at the end of the day. (a) transketolase (TRK); (b) triose phosphate isomerase (TPI); (c) phosphoenolpyruvate carboxylase (PEPC); (d) pyruvate kinase (PK); (e) phosphoglucoisomerase (PGI); (f) shikimate dehydrogenase (SKDH); (g) ADP glucose pyrophophorylase (AGPase); (h) fumarase; (i) uridine diphosphate-glucose pyrophosphorylase (UGPase); (j) phosphoglucomutase (PGM); and (k) glutamate dehydrogenase (GDH).

**Table 2:** Comparison of enzyme activities in mature leaves of Proteaceae species and in *Arabidopsis* grown in short days (4 h light) and long days (18 h light)

		PEPC	SKDH	PK	AGPase	PGI	Fumarase	TRK	Rubisco (maximal)	PGM	UGPase	TPI
Species												
* Banksia attenuata*		197	190	244	411	720	469	1944	2343	4077	3810	123447
* Banksia candolleana*		168	100	286	405	822	915	1542	2254	3109	3736	111966
* Banksia menziesii*		249	288	319	1639	1318	1425	4553	5652	5603	7905	230121
* Hakea flabellifolia*		258	261	241	832	2189	1602	3018	3349	7090	6137	165348
* Hakea neurophylla*		133	206	157	789	1670	816	3561	3089	7095	8412	195369
* Hakea prostrata*		218	314	273	1028	1857	1402	3665	4094	6712	8487	194482
Average		204	227	253	851	1429	1105	3047	3463	5614	6414	170122
* Arabidopsis* (SD)		590	638	605	532	1162	3368	4612	4536	6920	5900	137842
* Arabidopsis* (LD)		506	655	769	918	9045	5550	5872	6665	9045	7396	167678
Ratio*		0.34:0.40	0.36:0.35	0.42:0.33	1.60:0.93	1.43:1.91	0.33:0.20	0.66:0.51	0.39:0.76	0.81:0.62	1.09:0.87	1.23:1.01

The full data set for the Proteaceae species and for Arabidopsis is shown in Figs 6. Enzyme activities are expressed in nmol g^−1^ FW min^−1^. See text and Supporting Information Table S1 for abbreviations. ^*^Ratio between average value in Proteaceae species and that in *Arabidopsis* in extremely short days (4 h light; top row) or the maximum value in long days (18 h; bottom row light). AGPase, ADP glucose pyrophophorylase; FW, fresh weight; LD, long days (18 h); PEPC, phosphoenolpyruvate carboxylase; PGI, phosphoglucoisomerase; PGM, phosphoglucomutase; PK, pyruvate kinase; SD, short days (4 h); SKDH, shikimate dehydrogenase; TPI, triose phosphate isomerase; TRK, transketolase; UGPase, uridine diphosphate-glucose pyrophosphorylase.

### Ribosomal protein as a fraction of total protein

We compared the species and developmental changes in ribosome abundance with the corresponding changes in total soluble protein concentration (Fig. 2c,d). To do this, we estimated what proportion of the total leaf soluble protein was allocated to cytosolic and plastidic ribosomes. Firstly, we used our measured values for abundance of cytosolic 18S and plastidic 16S RNA (Fig. 2a,b), and literature values for the ribosome protein complement and the molecular weights of ribosomal proteins (TAIR10 *Arabidopsis* annotations) (Supporting Information Table S5) to estimate how much protein was allocated to cytosolic and plastidic ribosomes in a given species and stage of leaf development. This value was then divided by the corresponding value for total leaf soluble protein to estimate the proportion of the total protein that is assigned to a ribosome species (Supporting Information Table S6, Fig. 2d). Most ribosomal proteins are encoded by gene families. For the calculations we took the molecular weight of the most abundant member, as estimated from The Affymetrix GeneChip Arabidopsis ATH1 Genome Array transcript levels; the error introduced by this assumption will be small, as there are only small differences in molecular weight between family members. Our calculation assumed that the molecular weights of ribosomal proteins in the Proteaceae species are similar to those in *A. thaliana*; this assumption is supported by the conserved sequences of most ribosomal proteins (not shown).

Ribosomes represent around 3% of the total soluble protein pool in mature *A. thaliana* leaves (group a), rising to >10% in growing leaves (groups c, d) (Supporting Information Table S7). The latter value probably still underestimates the proportion of protein allocated to ribosomes in the cell division stage, when leaves are <1 mm in length (Baerenfaller *et al.* 2012). In the Proteaceae species, 2.5–8.0% of the total soluble protein is allocated to ribosomes in mature leaves, and a similar or slightly higher proportion in immature leaves (Fig. 2c,d). The lower levels of ribosomes per unit FW in the Proteaceae species are therefore not due to a major change in relative allocation of protein between ribosomes and other proteins for the mature leaves. Rather, it points to a different growth strategy. A low ribosome abundance can be expected to decrease the rate of protein synthesis, and hence the protein concentration and the rate of growth. This is especially evident for the plastidic ribosomes. In *A. thaliana*, the percentage of total soluble protein allocated to plastidic ribosomes was about 1.2 and 1.6% in leaves after maturation and in the late growth phase (groups a and b, respectively; Fig. 2d). A similar range was found in mature leaves of the Proteaceae species (1.0–3.8%, Fig. 2d). In young expanding *A. thaliana* leaves (group c), about 4% of total soluble protein was allocated to plastidic ribosomes (Fig. 2d), compared with only 0.7–2.7% in immature leaves from the Proteaceae species (Fig. 2d). These results point to a very low allocation of protein, and by inference, P to plastidic ribosomes during the earlier stages of leaf development in the Proteaceae species.

### Changes in rRNA levels in young and mature leaves of *A. thaliana* in P-deficient conditions

It is known that RNA levels, and by implication, ribosome abundance decrease in P-deficient *A. thaliana* (Hewitt *et al.* 2005). However, it is not known how P deficiency affects ribosome abundance along the leaf developmental gradient. We divided the leaves of P-replete and P-deficient *A. thaliana* plants into three groups: young leaves (<1–9 mm length), mid-state (9 mm to second largest) and mature leaves (largest to oldest leaves). Compared with P-replete plants, in P-deficient plants, cytosolic and plastidic rRNA abundance decreased by about 50 and 65%, respectively, in mature leaves, but they did not decrease significantly in young leaves of *A. thaliana* (Supporting Information Table S6). Total P in rRNA decreased by about 60% in mature leaves, but only by 10–15% in young leaves under P deficiency. For comparison, soluble protein levels decreased by about 25% in mature leaves, and 10% or less in young leaves (Supporting Information Table S6 and Fig. S1). Thus, P-limited *A. thaliana* maintained ribosome abundance, including plastidic ribosome abundance, in young leaves, and decreased ribosome abundance in mature leaves, accentuating the ribosomal developmental gradient observed in the *A. thaliana* rosette. This contrasts with the Proteaceae species, which did not show a high investment in ribosomes, and in particular plastidic ribosome abundance, in their immature leaves, pointing to contrasting strategies between the species.

### Total P pool and fraction of total P invested in rRNA

Total P concentration was determined in *A. thaliana* leaves grown in P-replete or P-deficient conditions (Supporting Information Table S7). Under P-replete conditions, *A. thaliana* leaves contained around 4.5 mg P g^−1^ DW, irrespective of their developmental stage. Under P-deficient conditions, *A. thaliana* retained a large amount of P in its youngest leaves, while the P concentration decreased to around 1 mg g^−1^ DW in the oldest leaves. The total concentration of P in leaves of the Proteaceae species was at least an order of magnitude lower, ranging from 0.12 to 0.86 mg g^−1^ DW. As in *A. thaliana* leaves grown under P-deficient conditions, young Proteaceae leaves contain more P than oldest leaves.

The proportion of P invested in rRNA followed a developmental gradient in *A. thaliana*, from 33 to 37% in its youngest leaves to 11–16% in the oldest ones. Perhaps surprisingly, this investment was totally insensitive to the P supply, which induced a decrease in total P concentration in the oldest leaves, thus indicating that *A. thaliana* allocated a fixed percentage of P to its translation machinery. Interestingly, the Proteaceae species did not show clear differences in the amount of P invested in their ribosomes in relation to the developmental stage of their leaves. Compared with *A. thaliana*, the total P in leaves of the Proteaceae species was at least an order of magnitude lower, and the proportion invested in rRNA was similar in older leaves or as little as half in younger leaves.

### Leaf chlorophyll

Overall, chlorophyll levels in mature leaves of Proteaceae species were similar to those in *A. thaliana* (Fig. 4a) Leaf chlorophyll concentrations were considerably (*P* ≤ 0.0001) lower in immature leaves of the Proteaceae species than in young expanding *A. thaliana* leaves (Fig. 4a). This reflects a delayed greening phenotype that is commonly seen in *Banksia* and *Hakea* species in the field (Fig. 1). Young expanding leaves have a yellow or reddish-brownish appearance in the studied *Hakea* species and are yellowish-brownish for the *Banksia* species under investigation (Fig. 1; Lambers *et al.* 2012b).

Interestingly, the Chl a/b ratio in mature leaves of the Proteaceae species was significantly (*P* = 0.005) lower (1.8–2.7) than for *A. thaliana* (3.5–4.2) (Table 1). Low Chl a/b ratios are typical for shade leaves, whereas the leaves of the Proteaceae species were sampled from plants growing in an open canopy in a Mediterranean climate, with peak light intensities over 2 mmol m^−2^s^−1^. These scleromorphic leaves are very thick, however; typically 0.5–0.8 mm (Table 1). Thick leaves tend to have shade-acclimated chloroplasts at their abaxial surface, for example in *Spinacia oleracea* (Nishio *et al.* 1993) and *Schefflera arboricola* (Lambers *et al.* 2008a), and this would explain the low Chl a/b ratio in the present Proteaceae species. The Chl a/b ratio was even lower (*P* ≤ 0.0001) in the immature leaves than in mature leaves (0.9–1.5, Fig. 2a), possibly reflecting a strong shading of these leaves by their trichomes and leaf pigments other than chlorophyll (Hughes *et al.* 2007).

### Starch and Glc6P

Starch levels in mature leaves of the Proteaceae species were measured in samples harvested at midday. The starch concentrations of mature leaves were two- to fourfold higher (*P* = 0.013) than those in leaves of *A. thaliana*, harvested at the end of the day. In a further experiment with the three *Banksia* species, starch levels showed no significant change between dawn and midday (Supporting Information Table S8), indicating that the mature leaves had accumulated a large amount of starch that was not being turned over. Starch levels were significantly (*P* ≤ 0.0001) lower in young expanding leaves than in mature leaves (Fig. 4b). This reflects the very low photosynthetic activity of young expanding leaves (Lambers *et al.* 2012b).

Glucose 6-phosphate plays a central role in metabolism, being a precursor for sucrose, starch and cell wall synthesis, a product of starch and sucrose degradation, and the starting point for glycolysis and the oxidative pentose phosphate pathway. It is also typically the most abundant phosphorylated intermediate in leaves (see, e.g. Hurry *et al.* 2000; Morcuende *et al.* 2007; Arrivault *et al.* 2009). Levels of Glc6P in mature leaves of the six Proteaceae species were similar to those of *A. thaliana* (*P* = 0.144) (Fig. 4c). This indicates that concentrations of phosphorylated intermediates are, in fact, higher on a protein basis in mature leaves of the Proteaceae species, compared with P-replete *A. thaliana*. Amino acid concentrations were three- to fourfold (average 3.2-fold) lower (*P* ≤ 0.0001) in mature leaves of Proteaceae species than in those of *A. thaliana*, especially when the latter were grown at longer photoperiods (Fig. 4d). Amino acid concentrations were also low in immature leaves, in contrast to *A. thaliana*, showing two-fold more amino acids in young leaves at similar expansion stage than in the expanding Proteaceae leaves (Supporting Information Table S4).

### Enzyme activities

Activities of Rubisco (Fig. 5) and a range of other enzymes (Fig. 6) in the Proteaceae species are shown in comparison with activities in *A. thaliana*. This comparison is summarized for the extreme day lengths in Table 2. The reliability of the measurements in the leaves of the Proteaceae species were validated by carrying out mixed extractions with *A. thaliana* rosettes (see Materials and Methods). The comparison made with *A. thaliana* in long day conditions (Fig. 5) showed significantly (*P* ≤ 0.01) lower concentrations in mature leaves of Proteaceae species than in *A. thaliana*. Because Rubisco N typically represents about 25% of total leaf N, and 50% of soluble protein, in sun-adapted leaves of C_3_ plants (Evans 1989), low Rubisco levels were expected in the Proteaceae species, based on their low total soluble protein concentrations (Fig. 3). Rubisco activities were, on average, about half of those in *A. thaliana* on a FW basis (Fig. 5, Table 2); this difference was smaller than that in leaf soluble protein concentration (Fig. 3). Rubisco activities were also lower on a leaf area basis than in *A. thaliana*, after accounting for the higher FW per unit leaf area in the Proteaceae species (Table 1).

Like Rubisco, a second Calvin–Benson cycle enzyme (transketolase, TRK; Fig. 6a, Table 2) also showed, on average, twofold lower (*P* = 0.0013) activities in mature leaves of the Proteaceae species than in *A. thaliana*. Similar activities were obtained for triose phosphate isomerase, TPI (Fig. 6b, Table 2) in the Proteaceae species and *A. thaliana* (*P* = 0.985). Levels of phosphoenolpyruvate carboxylase (PEPC; Fig. 6c, Table 2), and the dedicated glycolytic enzymes pyruvate kinase (PK; Fig. 6d, Table 2) and phosphoglucomutase (PGM; Fig. 6e, Table 2), were all significantly lower in mature leaves of Proteaceae species than in leaves of *A. thaliana* (*P* ≤ 0.01). Shikimate dehydrogenase (SKDH) levels were also significantly lower in mature leaves of the Proteaceae species than in *A. thaliana* (Fig. 6f, Table 2). It should, however, be noted that the enzyme activities showed a smaller difference between the Proteaceae species and *A. thaliana* than the difference in total soluble protein (Figs 3 & 6, Table 2).

Initial (data not shown) and total Rubisco levels in the Proteaceae species were significantly (*P* ≤ 0.0001) lower in young expanding leaves than in mature leaves (Fig. 5). The activities of two other Calvin–Benson cycle enzymes [TRK (Fig. 6a); TPI (Fig. 6b) ], were also significantly lower (*P* ≤ 0.0001) in young expanding leaves and rose strongly in mature leaves, although the changes were not as marked as for Rubisco. It should be noted that whereas Rubisco is unique to the Calvin–Benson cycle, TRK and TPI are also required at lower activities for carbohydrate breakdown *via* glycolysis and the oxidative pentose pathway. The key enzyme in starch synthesis (ADP glucose pyrophophorylase, AGPase) was also significantly (*P* = 0.0083) higher in mature leaves (Fig. 6g).

Other enzymes showed a reverse pattern. Activities of PEPC (Fig. 6c) and enzymes involved in glycolysis, for example PK (Fig. 6d), in organic acid metabolism (fumarase; Fig. 6h), and in amino acid biosynthesis (SKDH; Fig. 6f) were significantly (*P* ≤ 0.0001) higher in immature leaves than in mature leaves. The difference ranged between two- to threefold for PEPC and PK, and up to fivefold for SKDH. Shikimate dehydrogenase is required for the synthesis of aromatic amino acids, and of phenolic compounds that are derived from them (Vermerris & Nicholson 2006), and a high activity might account for the high concentration of the pigments referred to earlier (Fig. 1), assuming these pigments are of a phenolic nature (Close & Beadle 2003). Alternatively, its high activity might account for a vital role of SKDH in lignin biosynthesis in these highly scleromorphic leaves. Both simple phenolics and lignin affect the nutritional value of leaves (Jung & Fahey 1983). Uridine diphosphate-glucose pyrophosphorylase, which is involved in sucrose metabolism and cell wall synthesis, was high in both immature and mature leaves (Fig. 6i). Phosphoglucoisomerase (Fig. 6e) and PGM (Fig. 6j) are required for sucrose synthesis, starch synthesis and glycolysis. They showed similar activities in immature and mature leaves (*P* = 0.0.820 and *P* = 0.203, respectively). We also investigated glutamate dehydrogenase, which is involved in the release of ammonium during amino acid catabolism and is strongly induced under carbon starvation conditions (Gibon *et al.* 2006, 2009). Its activity was significantly (*P* = 0.005) lower in immature leaves than in mature leaves (Fig. 6k).

The general picture that emerges is that activities of enzymes involved in carbohydrate breakdown and growth were relatively high in immature leaves and much lower in mature leaves. Enzymes involved in photosynthesis followed the pattern seen for chlorophyll, and were low in immature leaves and much higher in mature leaves. Enzymes with functions in carbohydrate synthesis and breakdown showed approximately the same activities at both leaf stages.

### Changes in metabolic traits and rRNA abundance related to total soluble protein

**Figure 7:**
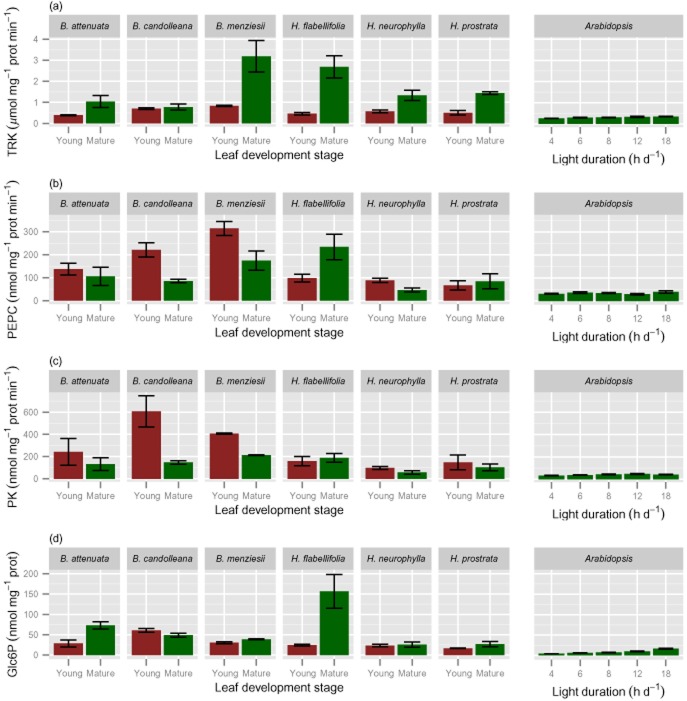
The level of selected enzymes (shown per unit fresh weight in Fig. 6) and a metabolite (shown per unit fresh weigh in Fig. 4) expressed per unit leaf protein (shown in Fig. 3). (a) transketolase, as example of photosynthetic enzymes; (b) phosphoenolpyruvate carboxylase and (c) pyruvate kinase as examples of respiratory enzymes; (d) glucose-6-phosphate.

Because leaves of Proteaceae species are much thicker, contain more structural dry matter and less protein than C_3_ species from mesic and nutrient-rich environments, like *A. thaliana* (Fig. 1, Table 1), the choice of unit is very important when comparing leaf traits. While all data displayed so far used leaf fresh weigh as the unit of reference, we also expressed the key parameters on a soluble protein basis. We consider that this best reflects the concentration per unit active cytoplasm. When expressed on this basis, compared with mature leaves of *A. thaliana* grown at an 18 h day length, mature leaves of the Proteaceae species (averaged for all six) contain similar or up to twofold higher levels of cytosolic and plastidic ribosomes (Fig. 2c,d), higher activities of Rubisco (2.5 times; Fig. 5c) and TRK (2.8 times; Fig. 7a) as examples of photosynthetic enzymes, higher activities of PEPC (3.0 times; Fig. 7b) and PK (4.6 times; Fig. 7c) as examples of respiratory enzymes, and higher levels of Glc6P (2.3 times; Fig. 7d). When young expanding leaves of the Proteaceae species are compared with young *A. thaliana* leaves, the leaves of *Banskia* species contain similar or higher levels of cytosolic ribosomes per unit protein, but two- to threefold lower levels of plastidic ribosomes. In the *Hakea* species, the levels of both cytosolic and plastidic ribosomes on a protein basis were lower than those in *A. thaliana*, with the exception of *H. prostrata*.

## Discussion

The present study significantly extends our recent paper (Lambers *et al.* 2012b) and reveals further novel findings on strategies that increase the efficiency of P utilization in Proteaceae species from P-impoverished habitats (Lambers *et al.* 2014b). The most fascinating result is the very low levels of rRNA, the major fraction of leaf organic P, in both young expanding and in mature leaves. We show that these Proteaceae species operate with very low levels of ribosomes, in particular plastidic ribosomes, at early stages in leaf development. This finding goes a very long way explaining the high PPUE in south-western Australian Proteaceae species (Wright *et al.* 2004; Denton *et al.* 2007; Lambers *et al.* 2012b). Secondly, we show a very marked ‘delayed greening’ in these Mediterranean species, and we can attribute a novel function for this process. This phenomenon has mainly been studied for tropical species (Kursar & Coley 1992a, 1992b; Cai *et al.* 2005), but is also reported for temperate species (Hughes *et al.* 2007). Thirdly, we provide evidence for very similar levels of a major P-containing small metabolite (Glc6P) to those found in P-replete *A. thaliana* leaves, and even higher levels if we express concentrations relative to soluble protein concentrations; thus taking into account the large fraction of the scleromorphic leaves with a large epidermal cell volume with a low metabolic activity. Fourth, we show that leaves from the Proteaceae species have low activities of several enzymes involved in carbon metabolism, in particular Rubisco and a set of enzymes involved in glycolysis; this would be expected based on their low total soluble protein concentration. This low protein concentration is accounted for by low abundance of ribosomes, which will restrict protein synthesis. Interestingly, low Chl a/b ratios in leaves exposed to high light intensities suggest internal shading of the thick leaves of Proteaceae species, while the relatively high levels of starch show that the plants are not carbon limited. We discuss these findings in the context of the high PPUE of Proteaceae from P-impoverished habitats, as well as the general relation between P investment in ribosomes and maintenance of a given protein concentration.

### Low investment of P in ribosomes

We found a considerably lower investment in ribosomes per unit FW in mature leaves of the Proteaceae species, and an even larger difference in their immature leaves, compared with P-replete and P-limited *A. thaliana* leaves. This low ribosome abundance may increase P-use efficiency in three ways. Firstly, it decreases investment of P in rRNA, which is often the major organic P-containing component in leaves (see Introduction). Secondly, lower ribosome abundance will decrease the rate of protein synthesis and cellular growth and, therefore, the demand for P in downstream processes like the synthesis of phospholipids and maintenance of adequate concentrations of free nucleotide and phosphorylated intermediates. Thirdly, the amount of P required in the translation machinery in immature leaves isdecreased by an altered developmental response of cytosolic and plastidic ribosome abundance.

The low ribosome abundance in immature leaves was largely explained by a low investment in plastidic ribosome abundance. It is striking that immature leaves are characterized by relatively high levels of cytosolic ribosomes, but very low levels of plastidic ribosomes, whereas mature leaves have similar levels of cytosolic and plastidic ribosomes. This indicates that protein synthesis in developing leaves of Proteaceae species occurs in two waves: a first wave is associated with cellular growth and establishment of the basic leaf structure, which requires cytosolic ribosomes, and a second wave is associated with chloroplast biogenesis, which requires both cytosolic and plastidic ribosomes. Chloroplast biogenesis requires large-scale protein synthesis in the cytosol, because most of the proteins in the plastids are encoded by the nucleus and are synthesized in the cytosol and immediately imported into the plastids to be incorporated into protein complexes that contain plastid- and nucleus-encoded proteins (Marín-Navarro *et al.* 2007; Cline & Dabney-Smith 2008).

In principle, the low ribosome abundance, the overall low total soluble protein concentration and slow growth could be explained in two ways: either lower ribosome abundance decreases the rate of protein synthesis and, hence both protein concentration and growth, or some other factor decreases growth and the protein concentration and, as a consequence, ribosome abundance. We consider, for two reasons, that the former is by far the most likely explanation. Firstly, ribosomes are required for protein synthesis (see Introduction). Secondly, rRNA abundance decreases more than the protein concentration for four of the six species for cytosolic rRNA and in all cases for plastidic rRNA.

In addition to net protein synthesis, rRNA is also required for protein turnover. We do not know the turnover rate of the photosynthetic proteins in these Proteaceae species in the field. Based on the measured levels of rRNA species and the total soluble protein concentration, we estimate that about 2.5–5.0% of the total soluble protein is allocated to ribosomes in mature leaves of the Proteaceae species, compared with about 3% in P-replete *A. thaliana*. The corresponding values for plastidic ribosomes were 1–3% and about 1.3%, respectively. The levels of ribosomes were even lower in mature leaves of P-limited *A. thaliana*. Assuming that these ribosomes are used with a similar intensity in all species, and that the majority of protein synthesis in these mature leaves is due to protein turnover, these results indicate that the rates of protein turnover in mature leaves of Proteaceae species are similar to or even marginally higher than those in mature leaves of greenhouse-grown *A. thaliana* plants. Given the intensity of stress to which Proteaceae species are exposed in the field, this is not surprising, although we also note that the proposed low-light environment inside the leaves of the Proteaceae species (see earlier) might decrease protein damage and consequently protein turnover (Raven 2012).

### Delayed greening

The low rates of photosynthesis (Lambers *et al.* 2012b) in young expanding leaves are associated with low levels of chlorophyll, starch, protein, Rubisco and other Calvin–Benson cycle enzymes. The young leaves were reddish or yellow, rather than green. This phenomenon of delayed greening has been described before for rainforest (Kursar & Coley 1992a; Close & Beadle 2003; Cai *et al.* 2005) and temperate species (Hughes *et al.* 2007). Delayed greening is considered a defence against herbivory (Kursar & Coley 1992b; Numata *et al.* 2004) as well as offering protection against high light intensities (Hughes *et al.* 2007). It has been proposed that delayed greening may result in a low protein level in younger leaves, which would decrease their nutritional value, while the red and yellow pigments may be defence compounds (Kursar & Coley 1992a). Phenolics have been observed in cotyledons of Proteaceae, where higher concentrations are associated with better defence (Hanley & Lamont 2001). In the Proteaceae species analysed in this study, soluble protein levels were, on average 1.5-fold higher in immature than in mature leaves. This developmental shift resembles that in *A. thaliana*, where soluble protein levels are around 1.3-fold higher in young leaves that have attained about half of their mature size than in mature leaves (Baerenfaller *et al.* 2012; Table 2). However, if the young expanding leaves had developed their photosynthetic machinery as happens in *A. thaliana*, the difference in total soluble protein would have been considerably greater, making the expanding leaves potentially more attractive for herbivores. While activities of most enzymes were low in young expanding leaves, these leaves showed relatively high levels of SKDH, which may be involved in the synthesis of the red and yellow pigments, assuming these are phenolics (Díaz *et al*. 1997; Peek *et al.* 2013). This comparison indicates that delayed greening is not necessarily associated with a decreased nutritional value *per se*, and emphasizes the potential importance of high levels of defence metabolites in the young leaves. These are presumably synthesized using imported carbon; the high levels of starch in mature leaves provide evidence that their synthesis is unlikely to be curtailed by carbon availability.

In *Quercus glauca*, delayed greening is considered important in the context of partitioning of N used for leaf expansion and N used for chloroplast development, two major sinks for N (Miyazawa *et al.* 2003). Ribosomes represent a high proportion of the total RNA and total protein in growing cells (Warner 1999; Raven 2012), including those in young leaves (Supporting Information Table S7; see also Detchon & Possingham 1972; Dean & Leech 1982; Baerenfaller *et al.* 2012). During leaf growth, both cell expansion and biogenesis of the photosynthetic apparatus require the synthesis of large amounts of protein (Miyazawa *et al.* 2003). Typically, in dicotyledonous species, leaf expansion and chloroplast biogenesis occur simultaneously (Detchon & Possingham 1972; Dean & Leech 1982; Baerenfaller *et al.* 2012). Our analyses of cytosolic and plastidic ribosome abundance in *A. thaliana* provide direct support for this model, showing that plastidic ribosomes are highly abundant in young expanding leaves, changing in parallel with the abundance of cytosolic ribosomes. The only exception, in wheat, is at the earliest stages of leaf development, when cytosolic ribosomes are more abundant (Dean & Leech 1982). An aspect that has not been considered previously is that delayed greening may also increase P-use efficiency and this might be the primary reason for the synthesis of non-plastidic pigments, which then can protect the leaves against high light in the absence of a mature photosynthetic machinery (see later).

Temporal separation of leaf expansion and establishment of the photosynthetic machinery lengthens the time needed for the establishment of a fully mature leaf, and especially the time until a leaf transitions from being a net importer to a net exporter of carbon. However, this is unlikely to be a major disadvantage in plants like the Proteaceae species, whose growth is limited by P, and which produce leaves that have a lifetime of 2–3 years. Their mature leaves accumulate large amounts of starch, which can be used to support growth of young leaves for an extended period before they develop a strong photosynthetic capacity and become self-supporting for carbon.

### Levels of Glc6P

Glucose 6-phosphate is a particularly abundant phosphorylated intermediate, and is typically about 20% of the fraction representing small P-containing metabolites (Hurry *et al.* 2000; Arrivault *et al.* 2009; Suzuki *et al.* 2012). Similar concentrations of this metabolite in leaves of Proteaceae species and in *A. thaliana* lead to rejection of our hypothesis that low leaf P concentrations are partly due to low levels of P-containing small metabolites. We suspect that the low concentration of Glc6P on a FW basis is due to a relatively small cytoplasmic volume occupied by photosynthetically active cells *per se*, and that the concentration in the cytoplasm and plastids of these cells may be quite high (Fig. 7). The response in these low-P adapted Proteaceae species differs markedly from that of *A. thaliana* and other species, like barley and spinach, where the level of Glc6P and other sugar phosphates drops strongly in P-limited plants (Brooks *et al.* 1988; Hurry *et al.* 2000; Zrenner *et al.* 2006; Morcuende *et al.* 2007). This decrease may be in part a direct consequence of a decrease in the cytoplasmic P pool, because a similar decrease occurs rapidly after feeding P-sequestering chemicals like mannose and glycerol to plants (Herold & Lewis 1977; Foyer *et al.* 1982; Prinsley & Leegood 1986; Leegood *et al.* 1988). Instead of economizing on P-containing metabolites, which would require higher enzymes levels (see Introduction), Proteaceae species economize on enzyme levels, corresponding with low rRNA levels. Because the levels of Glc6P represent only about 5% of total leaf P in these Proteaceae species, changes in Glc6P levels will make a far less significant contribution to PPUE than changes in rRNA abundance. An interesting implication of the relatively high Glc6P pool in these Proteaceae species is that the cytoplasmic P pool may not decrease as much as it does under P limitation in less well-adapted species.

### Low levels of enzymes associated with carbon metabolism to achieve high PPUE

Mature leaves of the Proteaceae species contain low levels of enzymes, including those for photosynthesis, and a low total soluble protein concentration. The enzyme activities were measured *in vitro* under optimal assay conditions. We have shown elsewhere that there is a good agreement between enzyme activities measured in our assays and protein abundance as determined by liquid chromatography tandem mass spectrometry (LC/MS-MS) (Piques *et al.* 2009). The low total soluble protein concentration is partly explained by a decreased level of enzymes in central metabolism. The observation that the total soluble protein concentration in Proteaceae species is five- to 10-fold less than in *A. thaliana* (Fig. 3), whereas the measured enzyme activities differ only two- to threefold or less (Table 2), indicates that other classes of soluble proteins may show an even larger decrease. This cannot be readily tested at this time, because we have so far not been able to obtain reliable measurements of metabolites when extracts from the present Proteaceae species are analysed by LC/MS-MS.

Our initial hypothesis, based on previous works in *A. thaliana* and crop plants, was that enzyme levels would remain high or even increase during leaf development in the leaves of the Proteaceae species. Our reasoning was that a low P availability would result in low levels of P-containing metabolites and cofactors, leading to enzymes being more strongly substrate limited *in vivo* and that this would be compensated by an increase in enzyme abundance. As proteins do not contain P, except when phosphorylated to be activated, this could provide a P-neutral strategy to compensate for lower levels of P-containing metabolites. Further, it might be expected that high levels of Rubisco and other Calvin–Benson cycle enzymes would be required to maximize photosynthesis in leaves that are exposed to the high light intensities in a Mediterranean climate where canopies are relatively open, as is the case for these Proteaceae species in the field (Fig. 1). Our results show that the Proteaceae species exhibit a very different strategy, decreasing investment in rRNA, decreasing protein synthesis and enzyme abundance while maintaining levels of Glc6P, and presumably other P-containing low molecular weight metabolites.

Four important considerations may explain why low levels of Rubisco, other Calvin–Benson cycle enzymes and soluble protein levels are compatible with rapid rates of photosynthesis per unit leaf P in leaves of the Proteaceae species. The first two considerations relate to the conditions under which photosynthesis may occur in the field in leaves of the Proteaceae species. Firstly, their leaves are rather thick, leading to shading of the chloroplasts at the abaxial side. This would also explain the very low Chl a/b ratio (Table 1), which is indicative of shade leaves (Lambers *et al.* 2008a). The especially low Chl a/b ratios in young leaves, may be due to additional shading by other pigments (Hughes *et al.* 2007) and leaf hairs. There is likely to be a gradient in Rubisco from the adaxial to the abaxial surface as well as a gradient in Chl a/b (Nishio *et al.* 1993; Vogelman *et al.* 1996). Secondly, leaves of these Proteaceae species do not necessarily carry out photosynthesis at the peak light intensities during midday. They typically decrease their stomatal conductance later during the morning (Lamont & Bergl 1991; Lambers *et al.* 2014a), and hence photosynthesize predominantly when the light intensity is half or less than that at midday. Further, while the leaves of the studied *Banksia* species are oriented fairly close to horizontal, those of the *Hakea* species are oriented vertically (Fig. 1), providing an additional reason why they do not photosynthesize at extreme midday irradiance levels. Both midday stomatal closure and the vertical orientation of the leaves maximize water-use efficiency (Farquhar & Sharkey 1982; Smith *et al.* 1998). Thus, despite high irradiance levels in their Mediterranean environment, the observed low total soluble protein concentrations and Calvin-Benson cycle enzyme levels can be understood, given the prevalent light intensities during photosynthesis. Thirdly (see earlier) is that the present Proteaceae species maintain high levels of Glc6P, and by implication, other phosphorylated intermediates, which will allow efficient operation of the Calvin–Benson cycle enzymes. Fourthly, and possibly the most decisive explanation for high rates of photosynthesis per unit leaf P relates to the P requirement of protein synthesis. Synthesis of proteins requires ribosomes (Rudra & Warner 2004, Warner, 1999), and rRNA is the major component of total cellular organic P (see Introduction), especially during the stage of leaf development linked with cellular growth and chloroplast biogenesis (Veneklaas *et al.* 2012).

The P content of rRNA represents 33 and 11% of the total P in young and mature leaves of P-replete *A. thaliana*, respectively (Supporting Information Tables S7 and S9). Surprisingly, this investment was independent of the P supply, despite the fact that P deficiency induced a decrease in the total P concentration in old leaves. Thus *A. thaliana* allocated a fixed percentage of its P to ribosomes, suggesting a tight regulation of the translation machinery in relation to P concentration of the leaves. When *A. thaliana* was grown at a low P supply, the P concentration was maintained in young growing leaves, and decreased in older and intermediate/expanding leaves. Ribosomes are used for growth mostly in young leaves, and in mature leaves are mostly used for maintenance. Wex would expect this strategy to be detrimental for *A. thaliana* for two reasons. Firstly, it is possible that oldest leaves under P deficiency do not contain enough ribosomes to allow maintenance of all cellular functions. Indeed, *A. thaliana* is loading around 60–65% of its ribosomes onto polysomes in the light under optimal growth conditions (Pal *et al.* 2013). This implies that the decrease of more than 50% in total ribosome amounts we observed in the oldest rosette leaves under P deficiency might not be compensated by a higher usage. It would lead to early senescence of these leaves as their maintenance cannot be ensured, thus explaining the well-known P deficiency phenotype. Secondly, the youngest *A. thaliana* leaves have similar amounts of ribosomes, irrespective of the P supply, but rosettes under such condition display reduced growth rates. Thus, probably a large proportion of these ribosomes in the young leaves may be in excess and constitute a wasted P pool. Such results point to an inefficient allocation of P resources by *A. thaliana* and might partly explain its low PPUE. In monocotyledonous species, leaf expansion and chloroplast biogenesis are separated in space and time (Dyer *et al.* 1971; Dean & Leech 1982; Pick *et al.* 2011). Separation of these processes in time might allow ribosomes to be initially engaged in the synthesis of proteins related to cellular growth, and subsequently in the synthesis of proteins involved in chloroplast biogenesis. This might allow the number of ribosomes to decreased, and improve the efficiency of N allocation (see earlier). It may also allow P investment in rRNA to decrease compared with plants where leaf expansion and greening occur simultaneously. In this case, we might expect this spatial and temporal separation of leaf expansion and chloroplast biogenesis to be especially advantageous under P-limited conditions. In agreement, monocots tend to have a higher PPUE than dicots, when grown under low-P conditions; this disappears when plants are grown at high P supply (Halsted & Lynch 1996).

### High leaf N:P ratios in leaves of Proteaceae species

The high N:P ratios observed in our study of Proteaceae (Table 1) are typical for species in similar environments (Lambers *et al.* 2010). They are not due to a high leaf [N], but to a very low leaf [P]; leaf [N] (Table 1) and leaf [protein] (Fig. 3) are actually relatively low. While low leaf N and total soluble protein concentrations do reflect the low availability of N in the natural environment of the species under investigation (Lambers *et al.* 2010), they may also reflect tight control of N uptake and metabolism when P is limiting, as found for other species (Rufty *et al.* 1993; de Magalhães *et al*. 1998; Gniazdowska & Rychter 2000). Even when grown at higher N supply, *H. prostrata* does not show high leaf [protein] (R. Jost, P.M. Finnegan & H, Lambers, unpublished results). While these species exhibit a low capacity to down-regulate their P uptake (Shane *et al.* 2004b; de Campos *et al.* 2013), their N uptake appears to be remarkably tightly controlled.

### Carbon allocation and storage

Unlike young expanding leaves, which show low rates of photosynthesis (Lambers *et al.* 2012b), mature leaves of these Proteaceae species accumulated considerable levels of starch, for example 125 *μ*mol hexose g^−1^ FW in *B. attenuata*, equating to the assimilation of 750 *μ*mol CO_2_ (Supporting Information Table S8). The rate of photosynthesis we measured for this species in the same environment (Lambers *et al.* 2012b) was 23 *μ*mol CO_2_ m^−2^s^–1^; at that rate, it would take about eight hours to accumulate the amount of starch we measured in this species, if none of the assimilates were exported at the same time. However, comparing starch levels at dawn with those at midday showed no significant difference (Supporting Information Table S8), and hence the rate of photosynthate export must have been close to the rate of production. Leaves are obviously not the site of long-term storage of reserves. Starch in *Banksia* species is predominantly stored in the vascular tissues of roots and trunks (Pate *et al.* 1990). During approximately three months in the wet season, these reserves would be important to support the carbon-demanding production and functioning of their cluster roots, which consume a major fraction of the daily produced photosynthates (Lambers *et al.* 2006). Later in the year, during the prolonged dry season when stomatal conductance of *Banksia* species declines (Lamont & Bergl 1991; Veneklaas & Poot 2003) and no cluster roots are produced (Lambers *et al.* 2006), the starch reserves would be required for maintenance of cellular structures allowing a negative carbon balance. The present photosynthesis measurements were taken in early summer (November–December), when temperatures are still mild and soil moisture levels high. Much of the starch would be needed to allow expansion of young and photosynthetically non-competent leaves, which typically occurs at this time of the year. However, Proteaceae species grow considerably slower than *A. thaliana*. For example, independent of P supply, *Banksia grandis* seedlings achieve a growth rate of 0.026 g g^−1^ day^−1^ (Barrow 1977), which is well over an order of magnitude less than that typically found for ruderal, low-LMA species (Lambers & Poorter 1992) and the rates measured for the *A. thaliana* plants used as a comparison in our study (0.28 and 0.56 mm^2^ mm^−2^ day^−1^ at a 12 and 18 h photoperiod, respectively (Gibon *et al.* 2009).

### Low leaf P concentrations, high rates of photosynthesis and high PPUE

The results in this and a recent paper (Lambers *et al.* 2012b) go a long way to explain the high PPUE of the investigated Proteaceae species. The mature leaf P concentration of these species in our study was is in the range of 0.15–0.29 mg P g^−1^ DW (Table 1; Lambers *et al.* 2012b). A basis for the following analysis is a canonical study in which barley (*Hordeum vulgare* L.) was grown in nutrient solution at a growth-limiting P supply, and analysed for total P concentration (0.9 mg P g^−1^ DW, and its distribution between the major P fractions in leaves: phospholipids (17%), P-containing metabolites (26%), nucleic acids (30%) and free Pi (26%) (Chapin & Bieleski 1982).

Firstly, as acknowledged before (Lambers *et al.* 2010), the high LDMC of approximately 0.55 g DW g^−1^ FW ‘dilutes’ leaf [P]. Thus, their very low leaf [P] on a DW basis, 10-fold lower than in plants showing an ‘adequate’ leaf [P] (Epstein & Bloom 2005), is reduced to a 1.8-fold difference when expressed on a FW basis. Secondly, we recently showed (Lambers *et al.* 2012b) that these Proteaceae species extensively replace phospholipids by galactolipids and sulfolipids during leaf development. This will very substantially reduce a fraction that represents approximately 17% of the P in leaves of P-limited barley (Chapin & Bieleski 1982) and a range of other species (Veneklaas *et al.* 2012). Thirdly, and most importantly, we found a decrease in ribosome abundance and, thence, the amount of P invested in rRNA. Compared with *A. thaliana*, rRNA abundance per unit FW was three to six times less in mature leaves of Proteaceae species, and up to 150-fold less in young expanding leaves. This massive decrease in a fraction that represents 30% of the P in P-limited barley leaves (Chapin & Bieleski 1982) will make a very large contribution to the 1.8-fold difference that is required to explain the difference in PPUE between Proteaceae species and plants that are not adapted to P-impoverished soils. Fourth, delayed greening may contribute to P-use efficiency. In mature leaves, the decrease in ribosome abundance closely tracked the decrease in total soluble protein concentration, while in young expanding leaves there was a particularly low investment in plastidic rRNA, which was accompanied by a delay in chloroplast biogenesis. In addition plastidic ribosome abundance on a protein basis was two- to threefold lower in young expanding leaves compared either with mature leaves of Proteaceae species or with young *A. thaliana* leaves. This is consistent with the proposal that delayed greening allows an increase in P-use efficiency by separating leaf growth and chloroplast biogenesis, thus allowing sequential use of the P invested in ribosomes. Plastidic ribosomes account for 25–36% of total ribosomes in a dicot leaf (Dyer *et al.* 1971; Makrides & Goldthwaite 1981), so delayed greening on its own might lead to a 25–36% decrease in P investment during this phase of leaf development.

The decreased allocation to two major leaf organic P fractions (phospholipids and nucleic acids) will increase P-use efficiency. The third major organic P fraction (P-containing metabolites) does not contribute to the high P-use efficiency, if all these metabolites show the same trend as Glc6P. The fourth major fraction of P in P-limited barley leaves is inorganic P. In eudicots, inorganic P is preferentially allocated to epidermal cells (Conn & Gilliham 2010). In *H. prostrata* (Shane *et al.* 2004a) and *B. attenuata* and *B. menziesii* (P. Clode & H. Lambers, unpublished), however, P is accumulated in mesophyll cells, as is the case in monocots (Conn & Gilliham 2010). To our knowledge, no systematic comparison has been made of the PPUE of dicots and monocots, with the exception of a study aimed at a comparison of C_3_ and C_4_ species (Halsted & Lynch 1996). Interestingly, Halsted & Lynch (1996) showed a greater PPUE in monocots. We do not know if Proteaceae species other than the three referred to earlier show a similar pattern of P allocation to mesophyll cells, instead of epidermal cells, but surmise that this might be an additional factor contributing to their high PPUE.

Do the data presented here and in our previous paper (Lambers *et al.* 2012b) also explain why rates of photosynthesis of the six Proteaceae species are relatively high on a leaf area basis, despite low leaf [P], leaf [N] and Rubisco activities? Because rates of photosynthesis were compared per unit leaf area, we should also compare other parameters on a leaf area basis. Protein levels per unit area were, on average, 1788 and 3783 mg m^−2^ for the six Proteaceae species and *A. thaliana*, respectively. The corresponding values for total Rubisco activities per unit leaf area were 35.7 and 21.1 *μ*mol m^−2^s^−1^, respectively. The six Proteaceae species assimilated 10–23 (average 16.7) *μ*mol CO_2_ m^−2^s^−1^ at the light intensity in the field in the morning which would have been saturating for photosynthesis (Lambers *et al.* 2012b). Conversely, *A. thaliana* leaves photosynthesize at about 7 *μ*mol CO_2_ m^−2^s^−1^ at the light intensity used for plant growth in the present experiments (Eckardt *et al.* 1997). Therefore, the Proteaceae species achieved, on average, a 2.3-fold higher rate of photosynthesis per unit leaf area with a 1.7 fold higher total Rubisco activity and only 47% of the amount of soluble protein per unit leaf area. This probably reflects an over-investment in Rubisco and use of some of the enzyme as a storage protein in *A. thaliana* (Quick *et al.* 1991; Irving & Robinson 2005). The *A. thaliana* plants used in this study were grown at a relatively low light intensity of 160 *μ*mol m^−2^s^−2^ and a high N supply; under such conditions there is likely to be substantial over-investment in Rubisco (Stitt & Schulze 1994). The Proteaceae species, which grew in their natural high-light environment, may operate with amounts of Rubisco much closer to the minimum required. This may be at least partly due to the lower levels of ribosomes, which constrain protein synthesis and the synthesis of large amounts of Rubisco.

### Concluding remarks

Young expanding leaves of the present six Proteaceae species function in a very different way from the fully mature leaves. Young leaves depend almost completely on import of carbon to sustain their expansion, while their chloroplast biogenesis is delayed. Delayed greening has previously been attributed to protection against herbivores and high light intensities (photo-protection), but here we suggest it is also a strategy to allow sequential use of rRNA for the synthesis of proteins required for the production of new cells and structural defence (sclerenchyma) and later for chloroplast maturation, thus decreasing the peak abundance of rRNA required during leaf development. We note that a similar strategy may also be relevant for N-limited plants, as ribosomes represent a major proportion of the total protein in young leaves. Following maturation, leaves contain massive amounts of sclerenchyma (Lambers *et al.* 2012b) and are very efficient at photosynthesis.

In both young expanding leaves and in photosynthetically competent mature leaves, there is a relatively low investment in protein, including Calvin–Benson cycle enzymes, which make up a large proportion of total soluble protein in leaves of C_3_ species. This is not necessarily disadvantageous, because leaves of the studied Proteaceae species predominantly fix carbon dioxide at levels of incident radiation well below midday levels, as a result of (1) decreased stomatal conductance during the middle of the day when transpiration rates would be high; (2) leaf angle; and (3) internal leaf anatomy. The lower levels of protein are at least partly a consequence of lower investment of P in rRNA. Taken together, the Proteaceae species thus optimize the use of both P and water. A low investment in rRNA would account, to a major extent, for the high PPUE, in addition to low levels of phospholipids (Lambers *et al.* 2012b) and possibly preferential allocation of P to mesophyll cells (Shane *et al.* 2004a) rather than epidermal cells, as is usual in other eudicots (Conn & Gilliham 2010).

Of the four main P fractions, that is rRNA, phospholipids, phosphorylated intermediates and free nucleotides, and free P, we have shown that the first, second and fourth are low in Proteaceae species grown in their natural low-P environment. We have no evidence that Glc6P, which is a major component of the pool of P-containing metabolites, that is phosphorylated intermediates and free nucleotides, is low, in contrast to findings for a range of other species exposed to low-P conditions (Veneklaas *et al.* 2012). From a systems perspective, this response represents a very effective strategy to maximize P-use efficiency. The low P allocation for rRNA will lead to a low rate of protein synthesis, and hence growth, matching it to the low P supply. Conversely, saving P by decreasing the concentration of P-containing intermediates of carbon metabolism would save P in that cellular component, but this would not decrease growth to match the P supply. In fact, low concentrations of P-containing intermediates might even lead to stress by slowing down metabolism. Therefore, the decrease in rRNA not only saves P in this cellular component, but also, by decreasing rates of protein synthesis and growth, further decreases the need for P in other pools, which are all located downstream of protein synthesis and growth. Maintaining high concentrations of Glc6P, and possibly other phosphorylated intermediates, on a protein basis will allow the enzymes, and hence the rRNA that is required for their synthesis, to operate effectively.

Future research will aim to explore if the P-efficiency traits in Proteaceae are desirable for crop plants, or if there are also unwelcome trade-offs. It will also be of interest to investigate whether differences in ribosome abundance contribute to competitiveness of wild species in natural and often challenging environments, and to investigate the regulatory mechanisms underlying these changes in ribosome abundance.

## Supporting Information

Figure S1 Rates of expansion of *Arabidopsis thaliana* rosette leaves; plants were grown in short days (8 h/16 h light/dark). **Table S1.** Recovery of enzyme activities. Powdered samples were prepared from a standard greenhouse-grown *Arabidopsis* (‘Arabidopsis’) and from a mix in equal parts of *Banksia attenuata*, *B. menziesii* and *B. candolleana* (‘*Banksia*-mix’). These powders were either extracted and assayed for enzyme activities separately, or combined in a 1:1 ratio (*Banksia*-mix + *Arabidopsis*) before extraction. The recovery of *Arabidopsis* enzyme activity was calculated as 100 × [Activity in (‘*Banksia*-mix + *Arabidopsis*)’ – (Activity in ‘*Banksia* mix' × 0.5) ] / (Activity in ‘*Arabidopsis’* × 0.5) × 100. This table also shows the abbreviations used for each enzyme, and assignment of each enzyme to a metabolic pathway as well as the dilution factor used for the assays and incubation times. **Table S2.** Threshold cycle values of external RNA standards in six Proteaceae species and in *Arabidopsis thaliana*. **Table S3.** Primers used for the determination of ribosome copy numbers in Proteaceae species and *Arabidopsis taliana*. List of the rRNA genes and spike controls (ArrayControl RNA Spikes; Applied Biosystems/Ambion, Darmstadt, Germany) use in qRT-PCRs. **Table S4.** Rates of expansion of *Arabidopsis thaliana* rosette leaves; plants were grown in short days (8 h/16 h light/dark). **Table S5.** Ribosome abundance in *Arabidopsis thaliana* and in six Proteaceae species. **Table S6.** Molecular weight of ribosomal proteins. **Table S7.** Percentage of investment of total phosphorus (P) in ribosomes. **Table S8.** Starch concentrations (*μ*mol glucose units g^–1^ dry mass) of fully expanded, mature leaves of *Banksia* species growing in their natural habitat. Leaves were collected either before dawn or later during the day, which started sunny, but with some clouds after 1130 h. Mean values and SE (*n* = 6). The time to produce this amount of starch was calculated from the concentrations as shown here, the leaf area per unit dry weight (Table 2) and published data on photosynthesis of plants grown in the same environment (Lambers *et al*. (2012b). **Table S9.** Phosphorus content in ribosomal RNA.
