# Supplementary material for: Low levels of ribosomal RNA partly account for the very high photosynthetic phosphorus-use efficiency of Proteaceae species
Source: Plant Cell Environ. 2013 Dec 23;37(6):1276–98. doi: 10.1111/pce.12240 (PMC4260170; doi:10.1111/pce.12240)
Supplement: Figure S1 — Rates of expansion of Arabidopsis thaliana rosette leaves; plants were grown in short days (8 h/16 h light/dark). Table S1. Recovery of enzyme activities. Powdered samples were prepared from a standard greenhouse-grown Arabidopsis (‘Arabidopsis’) and from a mix in equal parts of Banksia attenuata, B. menziesii and B. candolleana (‘Banksia-mix’). These powders were either extracted and assayed for enzyme activities separately, or combined in a 1:1 ratio (Banksia-mix + Arabidopsis) before extraction. The recovery of Arabidopsis enzyme activity was calculated as 100 × [Activity in (‘Banksia-mix + Arabidopsis)’ – (Activity in ‘Banksia mix' × 0.5) ] / (Activity in ‘Arabidopsis’ × 0.5) × 100. This table also shows the abbreviations used for each enzyme, and assignment of each enzyme to a metabolic pathway as well as the dilution factor used for the assays and incubation times. Table S2. Threshold cycle values of external RNA standards in six Proteaceae species and in Arabidopsis thaliana. Table S3. Primers used for the determination of ribosome copy numbers in Proteaceae species and Arabidopsis taliana. List of the rRNA genes and spike controls (ArrayControl RNA Spikes; Applied Biosystems/Ambion, Darmstadt, Germany) use in qRT-PCRs. Table S4. Rates of expansion of Arabidopsis thaliana rosette leaves; plants were grown in short days (8 h/16 h light/dark). Table S5. Ribosome abundance in Arabidopsis thaliana and in six Proteaceae species. Table S6. Molecular weight of ribosomal proteins. Table S7. Percentage of investment of total phosphorus (P) in ribosomes. Table S8. Starch concentrations (μmol glucose units g–1 dry mass) of fully expanded, mature leaves of Banksia species growing in their natural habitat. Leaves were collected either before dawn or later during the day, which started sunny, but with some clouds after 1130 h. Mean values and SE (n = 6). The time to produce this amount of starch was calculated from the concentrations as shown here, the leaf area per unit d [file pce0037-1276-SD1.zip › pce12240-sup-0003-tableS1-2,8.docx]

**Table S1.** Recovery of enzyme activities. Powdered samples were prepared from a standard greenhouse-grown *Arabidopsis* (‘Arabidopsis’) and from a mix in equal parts of *Banksia attenuata, B. menziesii* and *B. candolleana* (‘*Banksia*-mix’). These powders were either extracted and assayed for enzyme activities separately, or combined in a 1:1 ratio (*Banksia*-mix + *Arabidopsis*) before extraction. The recovery of *Arabidopsis* enzyme activity was calculated as 100 x [Activity in (‘*Banksia*-mix + *Arabidopsis*)’ – (Activity in ‘*Banksia* mix’ x 0.5)] / (Activity in ‘*Arabidopsis’* x 0.5) x 100. This table also shows the abbreviations used for each enzyme, and assignment of each enzyme to a metabolic pathway as well as the dilution factor used for the assays and incubation times.

| Enzyme | **Pathway** | | | | | | **Recovery of Arabidopsis enzyme activity in a mixed extract** | | **Final dilution of extracts for enzyme assays and incubation time** |
| --- | --- | --- | --- | --- | --- | --- | --- | --- | --- |
|  | Calvin-Benson cycle | Starch | Sucrose | Glycolysis | Organic acid | Amino acid synthesis |  |  |  |
| **Rubisco (initial)** |  |  |  |  |  |  | 84 |  | 500X – 45 sec |
| **Rubisco (maximal)** |  |  |  |  |  |  | 78 |  | 500X – 45 sec |
| **Transketolase (TRK)** |  |  |  |  |  |  | 98 |  | 4000X – 20 mins |
| **Triose phosphateiIsomerase (TPI)** |  |  |  |  |  |  | 87 |  | 20000X – direct |
| **ADP glucose pyrophosphorylase (AGPase)** |  |  |  |  |  |  | 130 |  | 1000X – 20 mins |
| **UDP-glucose pyrophosphorylase (UGPase)** |  |  |  |  |  |  | 106 |  | 5000X – 20 mins |
| **PEP carboxylase (PEPC)** |  |  |  |  |  |  | 114 |  | 2000X – 20 mins |
| **Pyruvate kinase (PK)** |  |  |  |  |  |  | 82 |  | 1000X – 20 mins |
| **Shikimate dehydrogenase (SKDH)** |  |  |  |  |  |  | 82 |  | 2000X – 20 mins |
| **Fumarase** |  |  |  |  |  |  | 78 |  | 10000X – 20 mins |
| **Phosphoglucose isomerase (total)** |  |  |  |  |  |  | 90 |  | 1000X – direct |
| **Phosphoglucomutase (PGM)** |  |  |  |  |  |  | 96 |  | 1000X – direct |
| **Glutamate dehydrogenase (NAD) (GDH)** |  |  |  |  |  |  | 115 |  | 500X – 20 mins |

**Table S2.** CT values of external RNA standards in six Proteaceae species and in *Arabidopsis thaliana*.

|  |  |  |  |  |  |  |  |  |  |  |  |  |  |  |  |  |  |  |
| --- | --- | --- | --- | --- | --- | --- | --- | --- | --- | --- | --- | --- | --- | --- | --- | --- | --- | --- |
|  |  |  |  |  |  |  |  |  |  |  |  |  |  |  |  |  |  |  |
|  |  |  |  | Mean |  |  |  |  |  |  |  | SD |  |  |  |  |  |  |
|  |  |  |  | Spike 1 | Spike 2 | Spike 3 | Spike 4 | Spike 5 |  |  |  | Spike 1 | Spike 2 | Spike 3 | Spike 4 | Spike 5 | R^2^ | Slope |
| Genus | Species | Leaf Stage | Copy number (log10) | 10.397 | 9.492 | 9.094 | 8.718 | 8.103 | R^2^ | Slope |  |  |  |  |  |  |  |  |
| Banksia | candolleana | mature | Ct Value | 18.254 | 20.641 | 21.371 | 22.625 | 25.063 | 0.980 | -0.339 |  | 1.1502 | 0.7467 | 0.8358 | 0.7407 | 0.8530 | 0.0111 | 0.0117 |
| Banksia | candolleana | young |  | 18.431 | 20.578 | 21.255 | 22.477 | 24.984 | 0.963 | -0.349 |  | 1.5933 | 0.8584 | 0.9525 | 0.8682 | 1.0096 | 0.0300 | 0.0268 |
| Banksia | menziesii | mature |  | 19.008 | 21.699 | 22.448 | 23.638 | 25.730 | 0.994 | -0.346 |  | 1.5896 | 1.5569 | 1.4771 | 1.6290 | 1.2997 | 0.0025 | 0.0125 |
| Banksia | menziesii | young |  | 16.694 | 19.289 | 20.203 | 21.403 | 23.605 | 0.994 | -0.336 |  | 0.2117 | 0.0165 | 0.0264 | 0.0922 | 0.1348 | 0.0017 | 0.0167 |
| Banksia | attenuata | mature |  | 20.369 | 22.726 | 23.232 | 24.605 | 27.131 | 0.970 | -0.341 |  | 0.2683 | 0.0233 | 0.1647 | 0.1367 | 0.2822 | 0.0115 | 0.0263 |
| Banksia | attenuata | young |  | 18.685 | 21.034 | 21.921 | 23.116 | 25.581 | 0.975 | -0.332 |  | 1.7143 | 1.2521 | 1.1826 | 0.7837 | 1.5040 | 0.0297 | 0.0097 |
| Hakea | neurophylla | mature |  | 18.766 | 21.564 | 22.175 | 23.378 | 26.048 | 0.973 | -0.318 |  | 1.0736 | 1.0147 | 1.0002 | 0.9034 | 1.1045 | 0.0301 | 0.0108 |
| Hakea | neurophylla | young |  | 18.839 | 21.421 | 21.930 | 23.228 | 25.822 | 0.972 | -0.332 |  | 0.5102 | 0.9787 | 0.9228 | 0.9609 | 0.8695 | 0.0096 | 0.0139 |
| Hakea | flabellifolia | mature |  | 21.105 | 24.252 | 25.160 | 26.367 | 28.568 | 0.995 | -0.311 |  | 3.2395 | 3.1394 | 3.3352 | 3.2878 | 3.3490 | 0.0034 | 0.0100 |
| Hakea | flabellifolia | young |  | 18.427 | 21.491 | 22.314 | 23.538 | 25.852 | 0.992 | -0.314 |  | 1.3086 | 1.0614 | 1.0461 | 1.0574 | 1.0760 | 0.0022 | 0.0096 |
| Hakea | prostrata | mature |  | 20.180 | 23.785 | 24.439 | 25.744 | 28.037 | 0.984 | -0.295 |  | 2.7275 | 3.0281 | 2.9065 | 2.9734 | 2.7598 | 0.0065 | 0.0034 |
| Hakea | prostrata | young |  | 18.315 | 21.619 | 22.490 | 24.034 | 26.101 | 0.996 | -0.296 |  | 1.0791 | 0.9793 | 1.0334 | 1.1816 | 1.1513 | 0.0025 | 0.0073 |
| Arabidopsis | thaliana |  |  | 18.054 | 20.945 | 21.842 | 23.196 | 25.482 | 0.995 | -0.312 |  | 0.1173 | 0.2241 | 0.0757 | 0.1102 | 0.0298 | 0.0008 | 0.0059 |

**Table S3.** Primers used for the determination of ribosome copy numbers in Proteaceae species and *Arabidopsis taliana*. List of the rRNA genes and spike controls (ArrayControl RNA Spikes; Applied Biosystems/Ambion, Darmstadt, Germany) use in qRT-PCRs.

**Table S4 and Figure S1.** Rates of expansion of *Arabidopsis thaliana* rosette leaves; plants were grown in short days (8 h/16 h light/dark).

**Table S5.** Ribosome abundance in *Arabidopsis thaliana* and in six Proteaceae species.

**Table S6.** Molecular weight of ribosomal proteins.

**Table S7.** Percentage of investment of total phosphorus (P) in ribosomes.

**Table S8.** Starch concentrations (μmol glucose units g^-1^ dry mass) of fully expanded, mature leaves of *Banksia* species growing in their natural habitat. Leaves were collected either before dawn or later during the day, which started sunny, but with some clouds after 11.30 am. Mean values and SE (n=6). The time to produce this amount of starch was calculated from the concentrations as shown here, the leaf area per unit dry weight (Table 2) and published data on photosynthesis of plants grown in the same environment (Lambers *et al.*, 2012b).

| Species | Time | | | Time to produce this amount of starch (h) |
| --- | --- | --- | --- | --- |
|  | 6 am | 9 am | 11.30 am |  |
| *Banksia attenuata* | 242±18 | 267±49 | 290±43 | 8.3 |
| *Banksia candolleana* | 214±51 | 305±92 | 283±38 | 14.4 |
| *Banksia menziesii* | 173±24 | 242±57 | 183±64 | 15.8 |

Table S9. Phosphorus content in ribosomal RNA.
